# Comprehensive Metabolomic Analysis of *IDH1*^*R132H*^ Clinical Glioma Samples Reveals Suppression of β-oxidation Due to Carnitine Deficiency

**DOI:** 10.1038/s41598-019-46217-5

**Published:** 2019-07-05

**Authors:** Satsuki Miyata, Kaoru Tominaga, Eiji Sakashita, Masashi Urabe, Yoshiyuki Onuki, Akira Gomi, Takashi Yamaguchi, Makiko Mieno, Hiroaki Mizukami, Akihiro Kume, Keiya Ozawa, Eiju Watanabe, Kensuke Kawai, Hitoshi Endo

**Affiliations:** 1 Department of Neurosurgery, Jichi Medical University, Tochigi, Japan; 2 Department of Biochemistry, Jichi Medical University, Tochigi, Japan; 3 Division of Genetic Therapeutics, Center for Molecular Medicine, Jichi Medical University, Tochigi, Japan; 4 Department of Pediatric Neurosurgery, Jichi Children’s Medical Center, Jichi Medical University, Tochigi, Japan; 5 Department of Medical Informatics, Center for Information, Jichi Medical University, Tochigi, Japan

**Keywords:** Cellular neuroscience, Cancer metabolism

## Abstract

Gliomas with *Isocitrate dehydrogenase 1* (*IDH1*) mutation have alterations in several enzyme activities, resulting in various metabolic changes. The aim of this study was to determine a mechanism for the better prognosis of gliomas with *IDH* mutation by performing metabolomic analysis. To understand the metabolic state of human gliomas, we analyzed clinical samples obtained from surgical resection of glioma patients (grades II–IV) with or without the *IDH1* mutation, and compared the results with U87 glioblastoma cells overexpressing *IDH1* or *IDH1*^*R132H*^. In clinical samples of gliomas with *IDH1* mutation, levels of D-2-hydroxyglutarate (D-2HG) were increased significantly compared with gliomas without *IDH* mutation. Gliomas with *IDH* mutation also showed decreased intermediates in the tricarboxylic acid cycle and pathways involved in the production of energy, amino acids, and nucleic acids. The marked difference in the metabolic profile in *IDH* mutant clinical glioma samples compared with that of mutant *IDH* expressing cells includes a decrease in β-oxidation due to acyl-carnitine and carnitine deficiencies. These metabolic changes may explain the lower cell division rate observed in *IDH* mutant gliomas and may provide a better prognosis in *IDH* mutant gliomas.

## Introduction

The number of patients with glioma is increasing annually with the aging of the global population in the US and Japan^1,2^. Because gliomas develop by infiltrating into normal brain tissue^3^, complete surgical excision is difficult. Therefore, efforts are underway to improve the degree of surgical excision using various modalities, including navigation and perioperative MRI. Advances have also been made in chemotherapy, immunotherapy^4^, molecular targeted agents^5^, intraoperative alkylating agents^6,7^, and radiotherapy. These treatments have improved the prognosis of glioma. However, even with these therapies, compared to other types of cancer, the median survival time for patients with glioblastoma remains poor at 21.4 months^8^.

The prognosis for patients with glioma with mutation in *Isocitrate dehydrogenase* (*IDH*) was reportedly better than that in patients without the *IDH* mutation (*IDH* normal)^9,10^. In patients with glioblastoma, the median survival in the *IDH* normal group was approximately 15 months, but surprisingly, the median survival in the *IDH* mutation group was approximately 31 months^10^. Clarifying the mechanism of this better prognosis may contribute to development of a new treatment strategy for gliomas.

*IDH* encodes an enzyme that converts isocitric acid to α-ketoglutaric acid (2-oxoglutaric acid; 2OG) and is categorized into three subtypes. IDH1 is localized in the cytoplasm, whereas IDH2 and IDH3 are localized in mitochondria. Mutation in *IDH1* is common in gliomas. Furthermore, mutation of the 132nd arginine to histidine (R132H) is a frequent *IDH1* mutation (*IDH1*^*R132H*^)^10^.

*IDH1*^*R132H*^ encodes an enzyme that produces D-2-hydroxyglutarate (D-2HG) from 2OG in the cytoplasm^11–15^. D-2HG competitively inhibits α-keto acid transaminase activity and alters metabolite levels in the tricarboxylic acid (TCA) cycle^16^. D-2HG is an oncogenic metabolite that inhibits several 2OG-dependent oxygenases, for example, DNA/RNA-modifying enzymes and JmjC domain-containing enzymes, which modify the epigenetic status of DNA and histones. Several CpG sites in genomic DNA are hypermethylated in glioma patients with *IDH* mutation^17^. Because the epigenetic changes occur in gliomas with *IDH* mutation via TET (Ten-eleven translocation) enzymes that catalyze a key step in the removal of DNA methylation, expression of several enzymes is altered, resulting in various metabolic changes^17,18^. On the other hand, D-2HG also inhibits ATP synthase and reduces ATP levels in *IDH1*^*R132H*^ mutant cells^19^, suggesting that this enzyme is a target of the growth–suppressive activity of D-2HG. The activity of a variety of enzymes, including those in the TCA cycle and ATP synthase, is thought to change through production of D-2HG due to the *IDH1* mutation.

In recent years, the metabolism of cancer cells has attracted much attention. Because the *IDH* mutation affects metabolism, mainly via the TCA cycle^16^, we conducted a comprehensive analysis of the metabolites of *IDH* mutant gliomas with mass spectrometry. In particular, we analyzed clinical glioma samples and compared their metabolism with that of cultured cells. We investigated the TCA cycle, lipid metabolism, amino acid metabolism, and energy metabolism to understand the mechanism of the better prognosis of *IDH* mutant gliomas.

In this study, we report that levels of D-2HG were significantly increased in the *IDH* mutant clinical samples similar to cultured glioma cells with *IDH* mutation, and that levels of intermediate metabolites such as those in the TCA cycle and ATP were altered, most likely through D-2HG activity. Moreover, β-oxidation was decreased only in the *IDH* mutant glioma samples due to reduced levels of carnitine. These results may explain why gliomas with *IDH* mutation have a better prognosis. Carnitine may be a better biomarker than D-2HG which is currently a well-known biomarker for *IDH* mutation. To our knowledge, this is the first report to show comprehensive metabolomic analysis using clinical samples of gliomas with *IDH* mutation.

## Results

### Metabolic profile of *IDH* mutant and *IDH* normal gliomas

**Figure 1:**
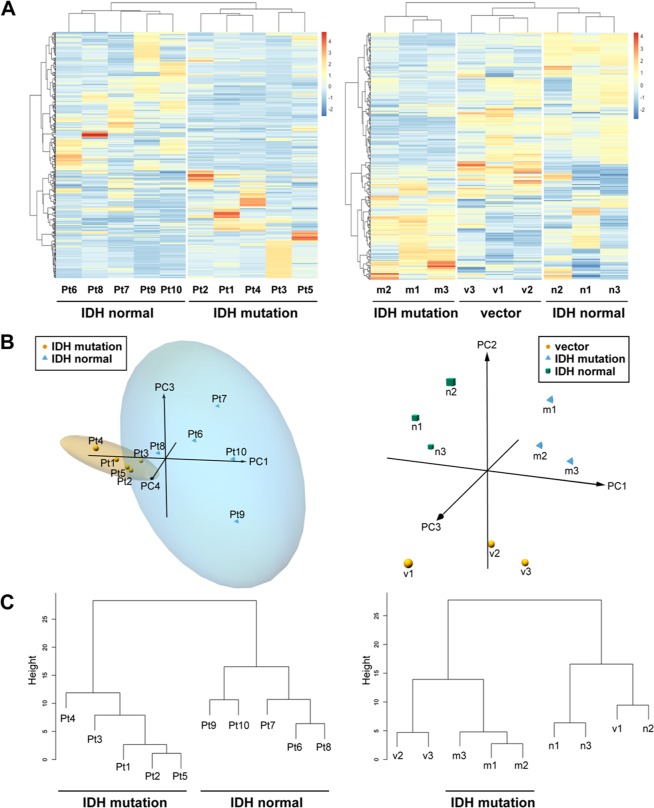
Heat map, Principal component analysis and Hierarchical clustering dendrogram in *IDH* mutant/normal gliomas. (**A**) The clustered heat map shows 396 biochemicals in lysates from five replicates each of IDH mutant glioma tissues (Pt1–Pt5) and normal *IDH* tissues (Pt6–Pt10) (Left). The heat map for U87 glioblastoma cells shows 261 biochemicals in triplicate lysates from cells expressing *IDH1* mutation (m1–m3), normal *IDH1* (n1–n3), or vector alone (v1–v3) (Right). (**B**) 3D plot of principal component analysis (PC Analysis) with fitted ellipses for the biochemicals in the major class category of The Human Metabolome Database (HMDB 3.0) (151 and 115 biochemicals in glioma tissues and U87 cells, respectively). PC1, PC3 and PC4 for glioma tissues (left, PC2 was omitted from the analysis as it did not show any relevant clustering information), and PC1, PC2 and PC3 for U87 cells (right) were plotted. In each group of glioma tissues, 95% confidence interval ellipsoid was illustrated. (**C**) Hierarchical clustering dendrogram obtained from the three principal components described in B.

We performed metabolomic analyses of transfected U87 glioblastoma cell lines that expressed mutant *IDH* or normal *IDH* and clinical glioma samples with or without *IDH* mutation using two methods, CE-TOFMS and LC-TOFMS. We detected 187 different metabolites (82 in cation mode and 105 in anion mode) in the U87 cells and 254 different metabolites (cations, 139; anions, 115) in the patient samples from CE-TOFMS analyses. Moreover, we detected 74 substance peaks (41 in positive mode and 33 in negative mode) in the U87 cells and 142 substance peaks (positive, 70; negative, 72) in the patient samples (Supplementary Dataset 1 and 2) from LC-TOFMS analyses. Then, we conducted a heat map analysis using these metabolome data. The clustered heat map shows 396 biochemicals in lysates from five replicates each of *IDH* mutant glioma tissues and *IDH* normal tissues (Fig. 1A, left). The clustered heat map for U87 cells showed 261 biochemicals in triplicate lysates from cells expressing *IDH1* mutation, *IDH1* normal, or vector alone (Fig. 1A, right).

One hundred fifty-one biochemicals for glioma tissues (Supplementary Dataset 2) and 115 biochemicals for U87 cells (Supplementary Dataset 1) were selected from The Human Metabolome Database (HMDB 3.0)^20^ and the principal component (PC) analysis for the biochemicals was conducted. The three-dimensional plot of PC using PC1, PC3, and PC4 for glioma tissues and PC1, PC2, and PC3 for U87 cells were shown in Fig. 1B. The PC analysis of *IDH* normal samples and samples with *IDH* mutation formed different PC clusters. This trend was further confirmed by hierarchical clustering dendrogram in Fig. 1C. Importantly, this trend could be observed even in samples from mutant *IDH* expressing U87 cells. This result clearly indicated that gliomas with *IDH* mutation had a different metabolic profile compared with that in *IDH* normal gliomas.

### Metabolic differences associated with *IDH* mutation

**Figure 2:**
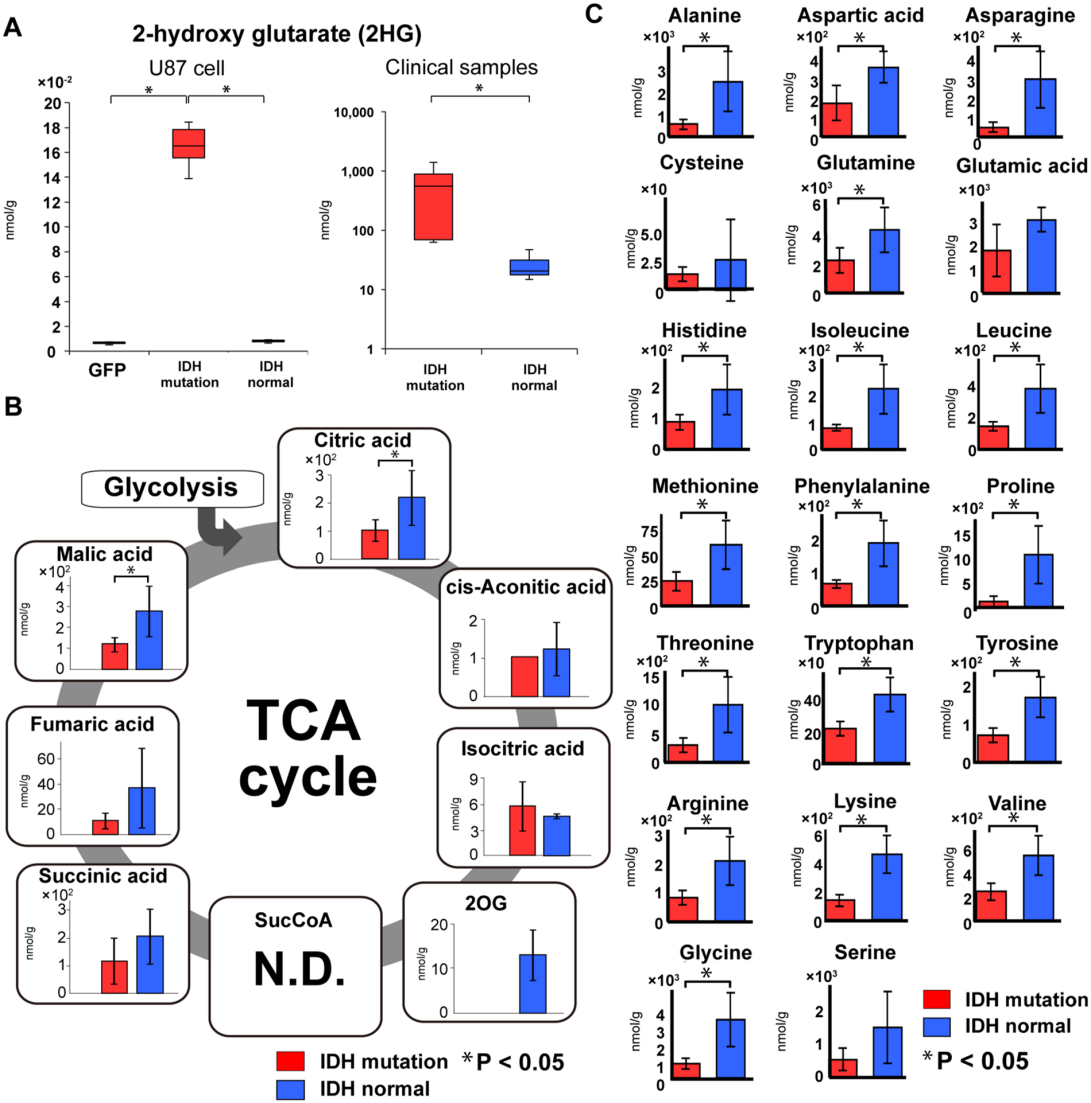
D-2-hydroxyglutarate, TCA cycle, and Amino acid in *IDH* mutant gliomas. (**A**) A box-whisker, the levels of D-2-hydroxyglutarate (D-2HG) were specifically elevated in *IDH* mutant glioma cells and clinical tumor specimens (red bars). For the U87 glioblastoma cell experiment, the vertical scale shows relative quantification based on the internal standard. (**B**) 2OG was considerably reduced in the *IDH* mutant glioma group (red bars) in clinical tumor specimens, and downstream intermediates of 2OG in the TCA cycle were also reduced. Each vertical scale shows the absolute quantification (nmol/g). (**C**) Amino acid production was reduced in the *IDH* mutant glioma group (red bars). The vertical scale for each amino acid shows the absolute quantification (nmol/g). Green bar, empty vector-transfected cells; red bars, *IDH1*^*R132H*^-transfected cells or *IDH1*^*R132H*^ mutant glioma tissues; blue bars, normal *IDH1*-transfected cells or normal *IDH1* glioma tissues; N.D., not detected; error bar, standard error of mean. *P < 0.05.

*IDH1*^*R132H*^ produces D-2HG from 2OG in the cytoplasm. D-2HG accumulated in gliomas with *IDH1* mutation in the previous reports^11–15^. Our experiments also showed that D-2HG levels were significantly increased in brain tumors bearing *IDH1*^*R132H*^ and U87 cells expressing *IDH1*^*R132H*^ (t-test, p < 0.05) (Fig. 2A). D-2HG is predicted to inhibit competitively α-keto acid transaminase, which results in inhibition of 2OG synthesis and inhibition of the TCA cycle^16^. Our experiments with U87 cells showed similar results as a previous study reporting that *IDH1*^*R132H*^ expression inhibits the production of 2OG and downstream intermediates in the TCA cycle (Supplementary Fig. S1)^21^. Experiments with the patient samples also showed similar results for intermediates in the TCA cycle (Fig. 2B). These results indicate that our sample preparation method and metabolomic data from the clinical samples are very reliable. The levels of cis-aconitic acid and isocitric acid, components of TCA cycle, in glioma samples with *IDH1* mutation were comparable with those in *IDH1* normal glioma samples compared with other components of TCA cycle. These biochemicals could be supplied by the anaplerotic reaction from pyruvate to oxaloacetic acid (OAA) by pyruvate carboxylase (PC) reaction not to stop TCA cycle^22^.

Non-essential amino acids are produced from or degraded to intermediate products of glycolysis and the TCA cycle, including 2OG, pyruvate, acetyl-CoA, fumaric acid, succinyl-CoA, and OAA. Therefore, the production of non-essential amino acids is affected considerably by metabolism in the TCA cycle. Interestingly, the amount of all 20 amino acids including essential amino acids was reduced in *IDH1* mutant brain tumors (Fig. 2C) and *IDH1*^*R132H*^-expressing U87 cells (Supplementary Fig. S2). These results were more pronounced in clinical samples than in the cell culture model. It is likely that the supply of essential amino acids is limited in tumors with *IDH* mutation or they are used up to compensate for the limited access of other metabolites such as non-essential amino acids.

**Figure 3:**
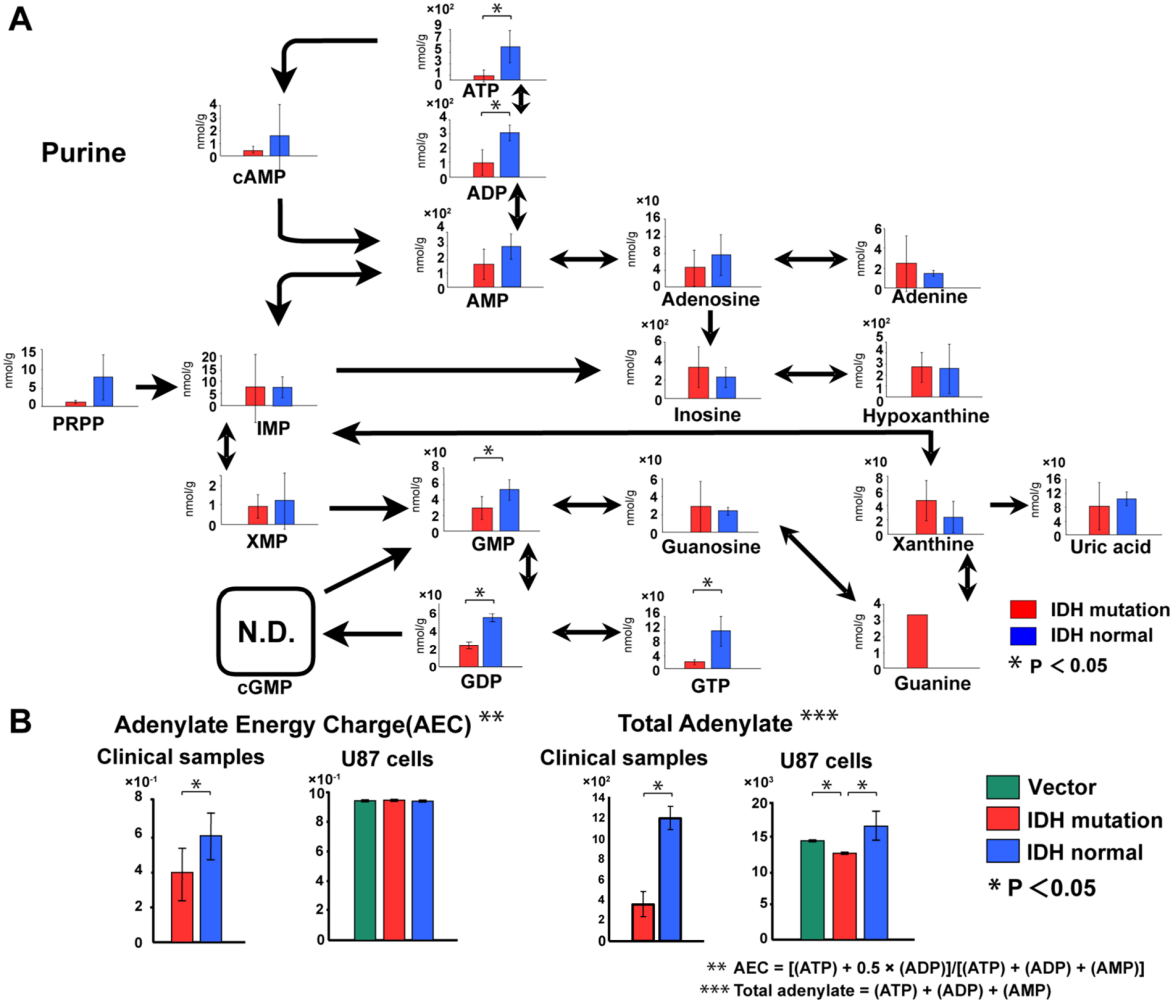
The purine pathway and energy production in *IDH* mutant gliomas. (**A**) The purine metabolic pathway was reduced in the *IDH* mutant glioma group (red bars). Each vertical scale shows the absolute quantification (nmol/g). (**B**) In the brain tumor tissue experiment, adenylate energy charge (AEC) and total adenylate were reduced. In the U87 glioblastoma cell experiment, we found no clear difference in AEC in cells expressing the *IDH* mutant (red bars), with each of the three groups having a value of 0.94. Adenylate production was reduced in *IDH* mutant glioma cells (red bars). Each vertical scale in the U87 cell graphs shows the calculated value relative to the internal standard. The vertical scales in AEC and total adenylate of brain tumors shows the calculated value. Green bars, empty vector-transfected cells; red bars, *IDH1*^*R132H*^-transfected cells or *IDH1*^*R132H*^ mutant glioma tissues; blue bars, *IDH1* normal-transfected cells or normal *IDH1* glioma tissues; N.D., not detected; error bar, standard error of mean. *P < 0.05.

The purine production pathway, which is responsible for nucleic acid production (ATP, ADP, AMP, etc.) and is closely related to energy production, was negatively affected in U87 cells expressing *IDH1*^*R132H*^ (Supplementary Fig. S3). Recently, Fu *et al*. showed that D-2HG directly inhibits ATP synthase and contributes to reduced levels of ATP in *IDH1*^*R132H*^ mutant cells^19^. Our experiments with patient samples also showed reduced levels of ATP in *IDH1* mutant gliomas (Fig. 3A). This result confirmed that a reduction in ATP production actually occurred in naturally occurring tumors, as well as the cell culture model that expressed mutant *IDH*. The adenylate energy charge (AEC) and total adenylate were reduced in *IDH* mutant gliomas from patients (Fig. 3B). In the cell line experiments, we found no difference in AEC among the groups, but total adenylate was slightly reduced in the *IDH* mutant cells (Fig. 3B).

### Reduced carnitine levels in *IDH* mutant glioma clinical samples

**Figure 4:**
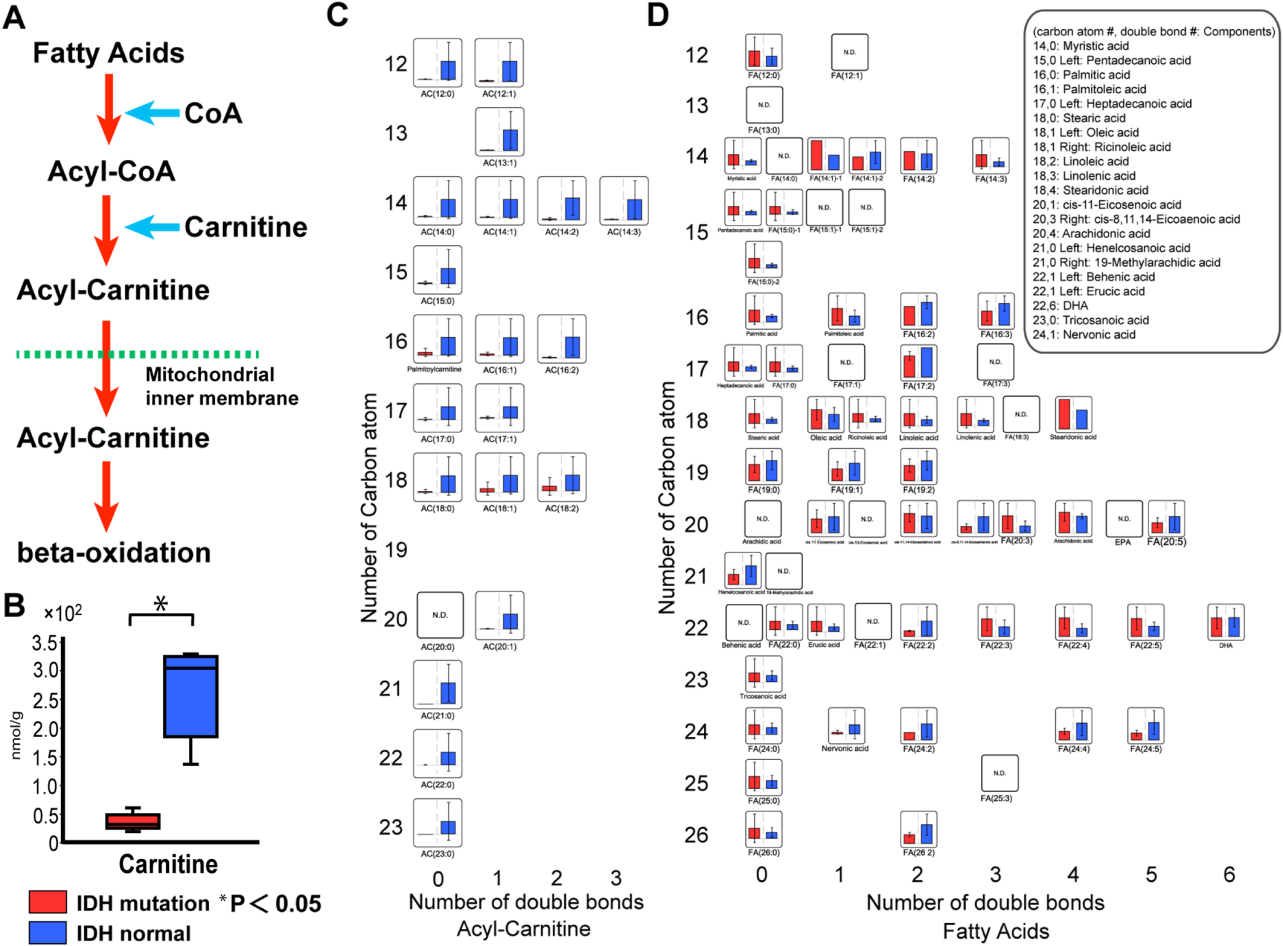
Analysis of carnitine and lipid metabolism in *IDH* mutation gliomas. (**A**) Schematic of the β-oxidation pathway: the lipid metabolism pathway. (**B**) Carnitine was reduced in the *IDH* mutant glioma group (red bars). The vertical scale shows the absolute quantification (nmol/g). (**C**) Acyl-carnitine was reduced in the *IDH* mutant glioma group (red bars). (**D**) Fatty acids were not obviously elevated in the *IDH* mutant glioma group (red bars). In graphs **C** and **D**, the vertical scales showed the number of carbon atoms. The horizontal axis scale shows the number of double bonds. The vertical scale shows the relative quantification based on the internal standard. Red bars, *IDH1*^*R132H*^ glioma; blue bars, normal *IDH1* glioma; N.D., not detected; error bar, standard error of mean. *P < 0.05.

Beta-oxidation takes place in a metabolic pathway that produces energy via oxidation of fatty acids, which bind carnitine in the mitochondrial intermembrane space and are converted to acyl-carnitine. They then cross the mitochondrial inner membrane and enter the mitochondrial matrix where they are converted to acetyl-CoA through β-oxidation. Acetyl-CoA is then used as a substrate in the TCA cycle and in other metabolic reactions (Fig. 4A). Interestingly, carnitine levels were significantly reduced in the *IDH* mutant group, especially in clinical samples (t-test, p < 0.05) (Fig. 4B and Supplementary Fig. S4). The degree of the difference in the level of carnitine between both groups was much clearer than that of D-2HG (Fig. 4B vs Fig. 2A). As a result of reduced levels of carnitine, levels of all acyl-carnitines were also reduced in the clinical brain tumor samples with *IDH* mutation (Fig. 4C), although fatty acid levels in *IDH* mutant samples were similar to those in normal *IDH* tumor samples (Fig. 4D). In the clinical brain tumor tissues, carnitine and acyl-carnitine were reduced strikingly in the *IDH1* mutant group, indicating suppression of β-oxidation. Because the levels of malonyl-CoA were not increased in glioma samples with *IDH1* mutation (Supplementary Dataset 2), fatty acid synthesis may not be activated. On the other hand, the levels of acyl-carnitine did not change in U87 cells expressing mutant *IDH1* and β-oxidation may not be suppressed in these cells (Supplementary Fig. S4). Although β-oxidation has been thought to be less efficient in the brain compared with other tissues, β-oxidation may be more efficient in *IDH1* normal than *IDH1* mutant gliomas. Markedly reduced β-oxidation activity in *IDH1* mutant gliomas may be involved in the reduction of energy production.

## Discussion

In this study, we performed a comprehensive metabolomic analysis of clinical glioma samples with and without *IDH* mutation and compared the results with an *IDH* mutant-expressing U87 glioblastoma cell line. We showed a significant increase in D-2HG and altered levels of intermediate metabolites such as those in the TCA cycle, energy production, amino acid production, and nucleic acid production in both the *IDH* mutant clinical samples and mutant *IDH*-expressing cells (Figs 2–4 and Supplementary Figs S1–S4). Most importantly, we could obtain statistically significant results from this metabolomic analysis although sample size in our current study using clinical samples is relatively small. Our detailed data analysis of biochemicals could clearly separate glioma samples with *IDH1* mutation from normal *IDH1* glioma samples (Fig. 1). This indicates that *IDH1* mutation causes metabolic reprogramming in cells and these metabolic changes may contribute glioma pathogenesis. Our metabolomics analysis of mutant *IDH* tumor samples indicated the significant reduction of carnitine and suppression of β-oxidation, which were the marked differences between the clinical samples and the cell lines (t-test, p < 0.05).

Mutant *IDH1*^*R132H*^ encodes an enzyme that aberrantly produces D-2HG from 2OG in the cytoplasm^11–15^. D-2HG is a competitive inhibitor of 2OG-dependent dioxygenases and affects downstream events such as DNA methylation and histone methylation. D-2HG is also predicted to competitively inhibit 2OG transaminase, which results in inhibition of 2OG synthesis, thereby inhibiting the TCA cycle^16^. Therefore, D-2HG is thought to affect many cellular processes through the activity of 2OG-dependent enzymes. In this study, we showed that the D-2HG level was significantly increased in tumor samples with *IDH* mutation and mutant *IDH*-expressing cells compared with control samples (Fig. 2A). 2OG and downstream metabolic intermediates in the TCA cycle were reduced in the mutatnt *IDH*-expressing glioblastoma cell line (Supplementary Fig. S1). This result matched well with a past study that used a different cell line^16^. Importantly, our metabolomic analyses using clinical tumor samples showed similar or more pronounced results (Fig. 2B), suggesting that our metabolomic analyses using clinical tumor samples with *IDH* mutation are highly reliable. Although a report was published that examined levels of D-2HG in clinical brain tumor samples with *IDH* mutation, this study is the first comprehensive metabolomic analysis of clinical glioma samples with *IDH* mutation^16^.

The decrease of ATP level was pronounced in glioma samples of *IDH* mutant group rather than cell culture system. Non-essential amino acids and acyl-carnitine as an energy source were also decreased in the clinical samples with *IDH* mutation. ATP production via lipid metabolism relies on β-oxidation. Carnitine plays an important role in the initiation of β-oxidation. Reitman’s study^16^ and our previous study^21^ using *IDH1*^*R132H*^-transfected cells did not show that carnitine levels were reduced. In this study, we showed that carnitine was significantly reduced in the clinical glioma samples with *IDH* mutation (Fig. 4B). Acyl-carnitine was also strikingly reduced in *IDH* mutant tissues, most likely because of carnitine deficiency.

**Figure 5:**
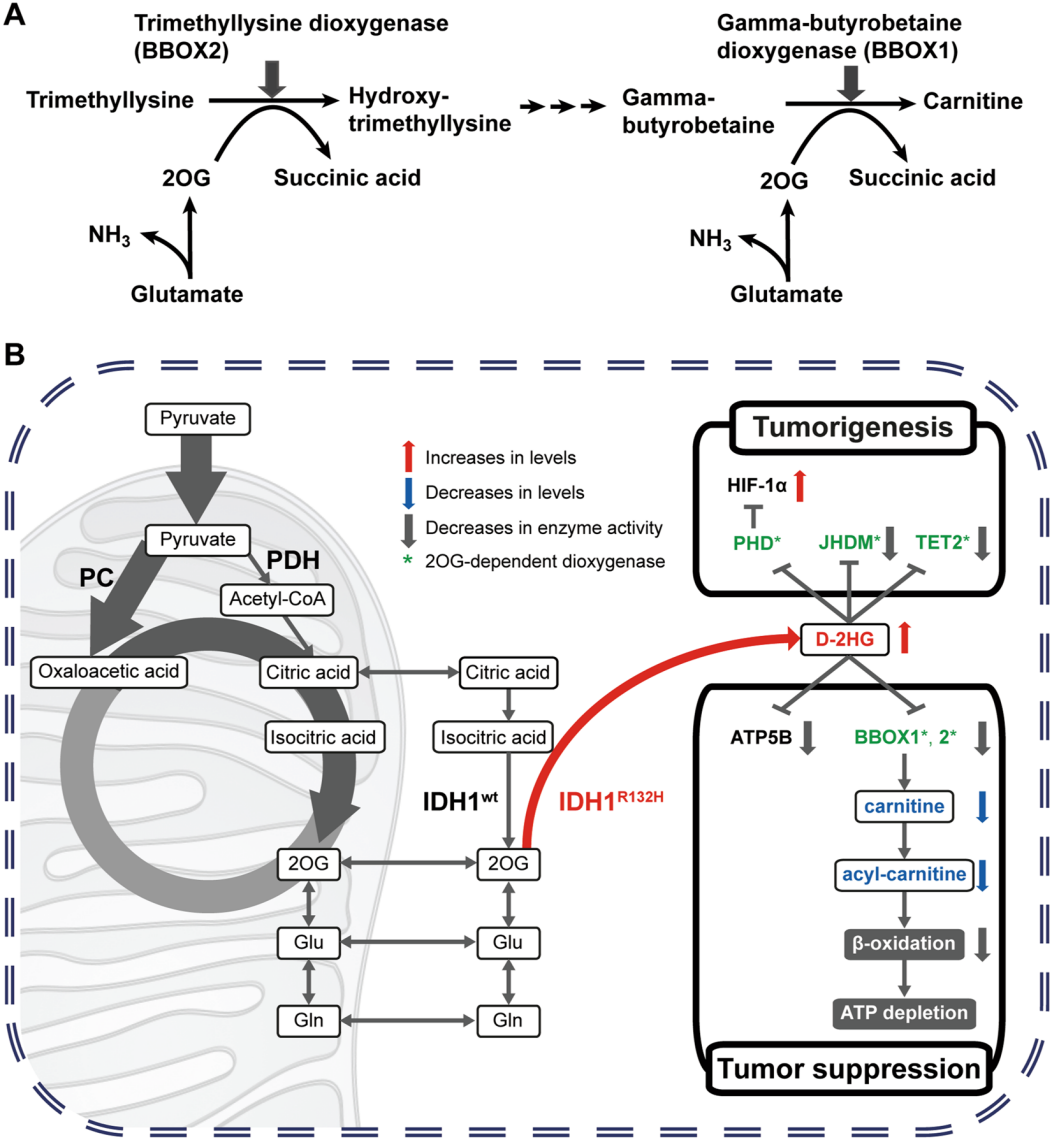
The hypothesis of the mechanism of *IDH* mutant gliomas. (**A**) 2OG-dependent dioxygenases in carnitine biosynthesis. There are two 2OG-dependent dioxygenases in the carnitine synthetic pathway. Carnitine is produced from gamma-butyrobetaine by gamma-butyrobetaine dioxygenase (BBOX 1) that is the last enzyme in the carnitine synthetic pathway. Trimethyllysine is converted into hydroxytrimethyllysine by trimethyllysine dioxygenase (BBOX 2) that is the first enzyme in the carnitine synthetic pathway. In these chemical reactions, 2OG acts as a cofactor. Reduced levels of 2OG in *IDH* mutant clinical tissues may lead to decreased carnitine synthesis through inhibition of these enzyme activities. (**B**) The proposed hypothesis for the mechanism of metabolism in *IDH* mutant tissues. The *IDH* mutation has two effects: a tumorigenic effect and a tumor suppressive effect. *IDH1* mutant gliomas produce D-2-hydroxyglutarate (D-2HG), resulting in inhibition of 2OG synthesis and downstream metabolic intermediates in the TCA cycle. As a tumorigenic effect, D-2HG produced by mutant *IDH* causes activation of HIF-1α through the inhibition of PHD (prolyl hydroxylases) activity and the direct inhibition of JHDM (Jumonji C-domain-containing histone demethylases) and TET2 activities. As a tumor suppressive effect, D-2HG inhibits ATP production through at least two ways. D-2HG directly inhibits ATP synthase activity by interaction with ATP5B (ATP synthase β subunit) and reduces ATP production. In addition, β-oxidation in clinical gliomas with *IDH* mutation is suppressed due to the reduction of carnitine levels. BBOX 1 and BBOX 2 in the carnitine synthetic pathway may be inhibited by high levels of D-2HG and lower levels of 2OG. Oxaloacetic acid could be supplied from pyruvate by pyruvate carboxylase (PC) as an anaplerotic reaction.

There are two 2OG-dependent dioxygenases in the carnitine synthetic pathway. Gamma-butyrobetaine dioxygenase (BBOX 1), which is the last enzyme in the carnitine synthetic pathway, produces carnitine using gamma-butyrobetaine and 2OG (Fig. 5A). Trimethyllysine dioxygenase (BBOX 2), which is the first enzyme in the carnitine synthetic pathway, converts trimethyllysine into hydroxytrimethyllysine. D-2HG may inhibit the activity of these enzymes and reduce carnitine levels, because D-2HG is a competitive inhibitor of 2OG-dependent dioxygenases^23,24^. In fact, some authors have speculated that BBOX 1 and BBOX 2 are inhibited by D-2HG^24^. Indeed, there is a report that BBOX 1 is inhibited by D-2HG at a relatively high IC_50_ *in vitro*^25^. Moreover, 2OG is a cofactor for both BBOX 1 and BBOX 2. The levels of 2OG in clinical glioma samples with *IDH* mutation are low, which may contribute to reduced levels of carnitine. Although carnitine synthesis mainly occurs in the liver, its synthesis may occur locally in the brain at lower levels^26,27^. The decrease in carnitine in *IDH* mutant tumors may result in suppression of β-oxidation, contribute to decreased ATP production, and relate to a better prognosis.

We found a discrepancy in carnitine and acyl-carnitine levels between the clinical glioma samples with *IDH* mutation and the *IDH1*^*R132H*^-transfected cells (Fig. 4 and Supplementary Fig. S4). Two explanations for this are possible. One is that sufficient amounts of carnitine may be supplied by the serum in the culture medium in the cell culture system, but not in *IDH* mutant tissues. We examined the effect of carnitine in the culture medium by growth rate of U87 cells. Supplementation of carnitine into the culture medium containing 10% dialyzed-fetal bovine serum (dialyzed-FBS) had a positive effect on cell growth of U87 cells. In addition, meldonium, a carnitine transporter and BBOX 1 inhibitor, supplemented to the 10% FBS-containing culture medium reduced proliferation of U87 cells (Supplementary Fig. S5). These data strongly suggested that the medium supplemented with FBS contains carnitine, which is important for proliferation and β-oxidation at least in part on cell culture system. The other is that 2OG was strongly decreased in the *IDH* mutant tissues, but only slightly decreased in the cell culture system (Fig. 2B and Supplementary Fig. S1). 2OG is replenished through the conversion from glutamine to glutamate, as well as the TCA cycle. A sufficient amount of 2OG may be synthesized from glutamine that is present in the culture medium in the cell culture system. However, a sufficient amount of glutamine may not be present in *IDH* mutant tissues. In fact, our metabolomic analyses showed that glutamine levels were reduced in *IDH* mutant tissues, but not in cell culture. As a result, we conclude that β-oxidation is decreased strikingly in the clinical glioma samples with *IDH* mutation. The supply of metabolites is likely altered locally in solid tumors, indicating the importance of investigations using clinical samples as in our current study.

The proposed hypothesis for the mechanism of metabolism in *IDH* mutant tissues is shown in Fig. 5B. The *IDH* mutation has two effects: a tumorigenic effect and a tumor suppressive effect. D-2HG produced by mutant *IDH* causes activation of HIF-1α through the inhibition of PHD (prolyl hydroxylases) activity and the direct inhibition of JHDM (Jumonji C-domain-containing histone demethylases) and TET2 activity^18,28–30^. These modulations of the activities are related to the tumorigenic effect. On the other hand, D-2HG inhibits ATP production through at least two mechanisms. D-2HG directly inhibits ATP synthase activity and reduces ATP production^19^. In addition to the reduction of ATP, we elucidated that β-oxidation in clinical gliomas with *IDH* mutation is suppressed due to the reduction of carnitine levels. BBOX 1 and BBOX 2, which are involved in carnitine synthesis, are 2OG-dependent dioxygenase and their activities may also be inhibited by D-2HG^24^. In addition to D-2HG production, because 2OG is maintained at very low levels in clinical gliomas with *IDH* mutation, BBOX 1 and BBOX 2 activities might be strongly inhibited. These are related to the tumor suppressive effect of *IDH* mutation.

For cell division, not only energy, but also amino acids and nucleic acids must be constantly supplied and are required at higher levels in cancer cells compared to normal cells. Our results suggest that cell division in *IDH* mutant gliomas is negatively affected and may explain why *IDH* mutant gliomas have a better prognosis. Pyruvate kinase isoform M2 (PKM2) is glycolytic enzyme catalyzing the conversion of phosphoenolpyruvate (PEP) to pyruvate. Serine binds to and activates PKM2, and that PKM2 activity in cells is reduced in response to serine deprivation^31^. Therefore, serine is related to glycolysis and the activity of glycolytic reaction affects the production of biochemicals. Methionine is related to DNA and RNA methylation^32^. Our study showed that serine, glycine, and methionine were decreased in gliomas with *IDH* mutation (Fig. 2C). The decrease of serine resulted in the suppression of folate metabolism and glutathione production. Folate is related to cell division and glutathione is related to oxidative stress. These results may also link to the better prognosis of gliomas with the *IDH* mutation (Supplementary Fig. S6).

The main purpose of this study was to understand the change of metabolism in clinical glioma samples. Thus, we just used U87 cell culture model as reference for some aspects such as D-2HG production because it does not sometimes reflect aspects of clinical samples. Furthermore, we verified the appropriate size of the clinical samples for the number of detected metabolites using MetSizeR^33^ and PASS16 (NCSS LLC, Kaysville, USA). It was presumed that this clinical study was needed to more samples (Supplementary Fig. S7). Collection of glioma clinical samples with *IDH* mutation is not easy due to low incidence of patients with this mutation in only one hospital. We will collect more samples, conduct metabolomic analysis, and further confirm our current results in the future. Regarding to the carnitine in the group sample sizes of 5 and 5, 70.4% power was calculated even when considering the Bonferroni’s correction for multiple testing, alpha = 0.00033 (Supplementary Table S1). Thus, regardless of the small sample size, it is thought that carnitine is sufficiently reliable.

In summary, we obtained a large amount of information by conducting metabolomics on *IDH* mutant glioma cells and clinical tissues. Our results demonstrate that, in *IDH* mutant gliomas, (1) the TCA cycle is inhibited, (2) β-oxidation is suppressed, especially in clinical samples due to carnitine deficiency, (3) energy production is reduced, and (4) molecules required for cell division (amino acids and nucleic acids) are reduced. These results may explain the mechanism for the better prognosis of gliomas with *IDH* mutation. We also propose that carnitine may be a better biomarker for *IDH* mutant gliomas than D-2HG. The carnitine synthesis pathway might become a therapeutic target for *IDH* normal glioma with poor prognosis.

## Methods

### Cell line

The human glioblastoma cell line U87 was obtained from ATCC and cultured as described previously^21^. U87 cells do not harbor the *IDH* mutation.

### Specimens from patient’s brain tumors

We obtained specimens from 10 patients who underwent surgery for glioma between 2006 and 2012 at Jichi Medical University. The clinical samples were used after obtaining informed consent from the patients and approval from the Jichi Medical University Ethical Board (approval number 11–31). This study was performed in accordance with the ethical standards of the 1964 Declaration of Helsinki, as well as the GCP guidelines. The tumor samples were collected during surgery, immediately frozen in liquid nitrogen in the operating room, and stored at −80 °C until metabolomic analysis. No anticancer drugs or medications (mercaptopurine, fluorouracil, etc.) that could modify the metabolism were administered prior to surgery. Patient clinical information is listed in Supplementary Table S2 and Fig. S8). Histopathology was performed in the Department of Pathology of the University. All tumors were classified according to the current WHO classification of tumors of the CNS^34^.

### Plasmids and transfection

Plasmids expressing normal IDH1 or IDH1^R132H^ under the control of the CMV promoter were described previously^21^. The plasmid was transfected into the U87 glioblastoma cell line using Lipofectamine 2000 (Invitrogen). We confirmed the expression of IDH1 by western blotting as described previously^21^.

### Detection of mutations in *IDH1* and *IDH2*

Total DNA was extracted from U87 cells and tumor samples using the DNeasy Blood & Tissue Kit (Qiagen). A DNA fragment of *IDH1* or *IDH2* was amplified by PCR with a pair of primers as described^10^, and mutations were confirmed by direct sequencing.

### Metabolomic analysis

Twenty-four hours after transfection, to inactivate enzymes, triplicate wells of U87 cells were quenched with methanol containing an internal standard (Solution ID: H3304-1002, Human Metabolome Technologies, Tsuruoka, Japan). Next, the cells were scraped off and stored at −80 °C until analysis. The number of transfected U87 cells for analysis is described in Supplementary Table S3A. The amounts of tumor samples that were used for analyses are described in Supplementary Table S3B. Tumor samples were quenched with 1500 μl acetonitrile and 20 μl D-camphor-10-sulfonic acids as an internal reference standard, and were homogenized on ice.

We normalized the data using sample weight for clinical samples and the number of cells for cultured cells. As the statistical test to compare the two groups, we use two-tailed Welch’s t-test. The Welch’s t-test is used when two samples have unequal variances (the violation of the homoscedasticity assumption) but fit to the normal distribution. In case of the violation of the normality assumption, the Mann-Whitney test was performed. The fitness to a normal distribution was examined before Welch’s t-test. Bonferroni adjustment was performed to correct P value for the multiple comparison.

Furthermore, to extract the candidates of biochemical compounds that attribute to either *IDH1* normal or *IDH1* mutant, the principal component analysis was performed after all data values were normalized with mean 0 and variance 1 by using a peak value of each compound to fit a normal distribution.

### Mass spectrometry

#### Capillary electrophoresis time-of-flight mass spectrometry (CE-TOFMS)

Metabolomics were measured and data were processed with an Agilent CE-TOFMS system (Agilent Technologies) equipped with a fused silica capillary with an internal diameter of 50 µm × 80 cm. Measurement of extracted metabolites in both the positive and negative modes was performed by using a commercially available electrophoresis buffer. Alignment of detected peaks was performed according to the mass-to-charge ratio (m/z) value and normalized migration time (Supplementary Table S4A). The glycolysis system, pentose phosphate cycle, TCA cycle, purine metabolic pathway, pyrimidine metabolic pathway, nicotinic acid, nicotinamide metabolic pathway, and various amino acid metabolic pathways were analyzed. We then performed the relative quantification of these data with the internal reference standard. We also performed measurements by adding the absolute quantitative value for 116 metabolites (Supplementary Dataset 1 and 2).

#### Liquid chromatography time-of-flight mass spectrometry (LC-TOFMS)

The positive and negative modes were performed using an Agilent 1200 series Rapid Resolution LC system SL (Agilent Technologies) equipped with an Octa Decyl Silyl analytical column of 2 mm × 50 mm and 2 µm particle size (Supplementary Table S4B).

Metabolome measurements were carried out by a facility service at Human Metabolome Technology Inc. (Tsuruoka, Japan).

### Data processing and analysis

The detected peaks were automatically extracted using the automatic integration software MasterHands ver.2.9.0.9 (Keio University, Tokyo, Japan), and the m/z, peak area as the peak information, migration time (MT) with CE-TOFMS, and retention time (RT) with LC-TOFMS were obtained. The obtained peak area was converted to the relative peak area with the following formula: Relative peak area = target peak area/ (internal reference standard peak area × sample volume).

The detected peaks were compared with all substances registered on the Human Metabolome Technologies Metabolic Substance Database based on the m/z and MT or RT values. The error tolerance for the search was ± 0.5 min for MT or RT and ± 10 ppm for m/z (Supplementary Dataset 1 and 2). The metabolome pathway mapping is shown as a bar graph with the obtained peaks. Abbreviations used in figures and tables are shown in Supplementary Table S5.

### Heat map, PCA, and hierarchical dendrogram

All analysis was performed using R^35^ (Version 3.4.3) with R Studio^36^ (Version 1.1.383). Heat map was generated using the R package “pheatmap” under Euclidean distance with ward. D2 linkage method for each row and column. Blue color represents lower concentration; red color represents higher concentration. PCA was performed using the R “prcomp” function, and the 3D plot was created using the R package “pca3d”. The cluster for hierarchical dendrogram was calculated using the R “hclust” function under Euclidean distance with ward. D2 method.

### The adenylate energy charge

The adenylate energy charge (AEC) and total adenylate, which serve as indicators of the energy status inside the cell, were obtained as follows: AEC = [(ATP) + 0.5 × (ADP)]/ [(ATP) + (ADP) + (AMP)]; total adenylate = [(ATP) + (ADP) + (AMP)].

## Supplementary information

Supplementary Information

Supplementary Dataset 1

Supplementary Dataset 2

## Data Availability

The datasets generated during and/or analyzed during the current study are available from the corresponding author on reasonable request.
