# Supplementary material for: Comprehensive Metabolomic Analysis of IDH1R132H Clinical Glioma Samples Reveals Suppression of β-oxidation Due to Carnitine Deficiency
Source: Sci Rep. 2019 Jul 5;9:9787. doi: 10.1038/s41598-019-46217-5 (PMC6611790; doi:10.1038/s41598-019-46217-5)
Supplement: Supplementary file 1 — Supplementary Information [file 41598_2019_46217_MOESM1_ESM.pdf]

Comprehensive Metabolomic Analysis of *IDH1*^{R132H} Clinical Glioma Samples Reveals Suppression of β-oxidation Due to Carnitine Deficiency

Satsuki Miyata[1*], Kaoru Tominaga[2*], Eiji Sakashita[2], Masashi Urabe[3], Yoshiyuki Onuki[1], Akira Gomi[4], Takashi Yamaguchi[1], Makiko Mieno[5], Hiroaki Mizukami[3], Akihiro Kume[3], Keiya Ozawa[3], Eiju Watanabe[1], Kensuke Kawai[1] and Hitoshi Endo[2*]

1. Department of Neurosurgery, Jichi Medical University, Tochigi, Japan
2. Department of Biochemistry, Jichi Medical University, Tochigi, Japan
3. Division of Genetic Therapeutics, Center for Molecular Medicine, Jichi Medical University, Tochigi, Japan
4. Department of Pediatric Neurosurgery, Jichi Children's Medical Center, Jichi Medical University, Tochigi, Japan
5. Department of Medical Informatics, Center for Information, Jichi Medical University, Tochigi, Japan

Address for correspondence: Satsuki Miyata (smiyata@jichi.ac.jp), Kaoru Tominaga (tominaga@jichi.ac.jp) and Hitoshi Endo (hendo@jichi.ac.jp)

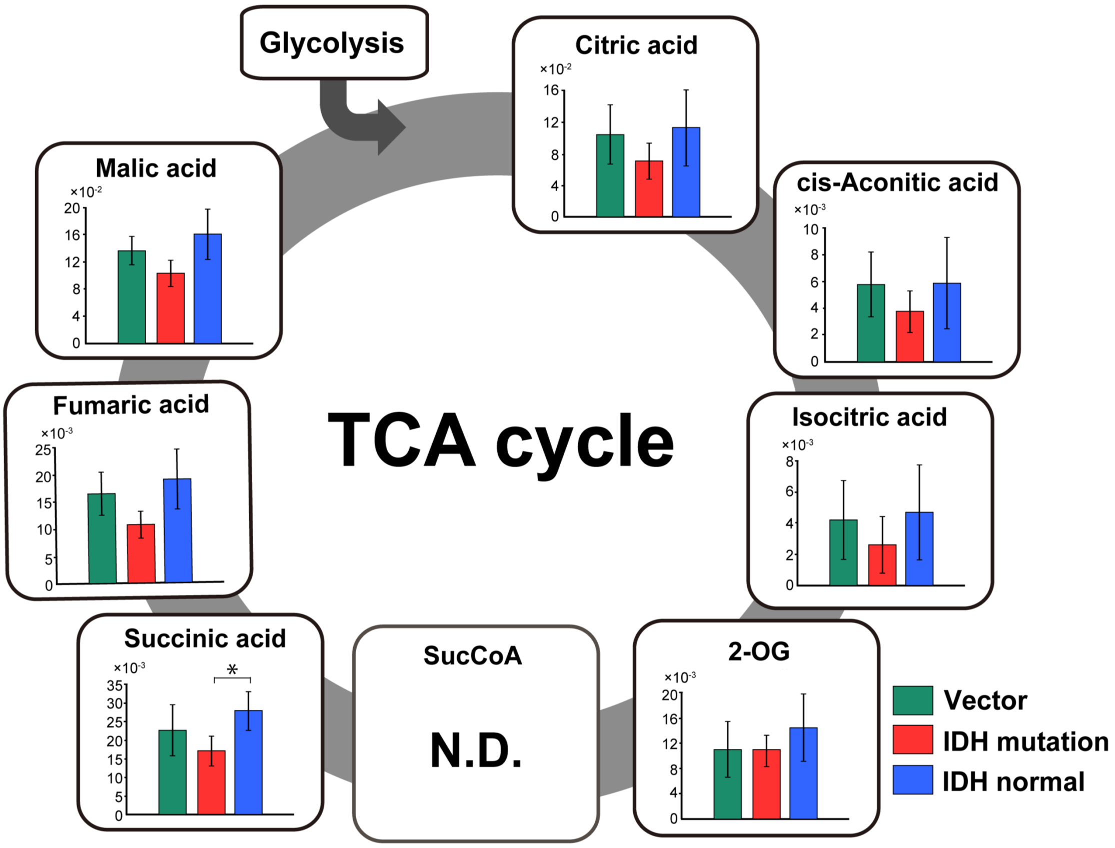

Supplementary Figure S1. TCA cycle analysis in the cell experiment.

The TCA cycle was reduced in the *IDH* mutant glioma cell line (red bars). Each vertical scale shows the relative quantification based on the internal standard. Green bars, empty vector-transfected cells; red bars, $IDH1^{R132H}$-transfected cells; blue bars, normal *IDH1*-transfected cells; N.D., not detected; error bar, standard error of mean. *P < 0.05.

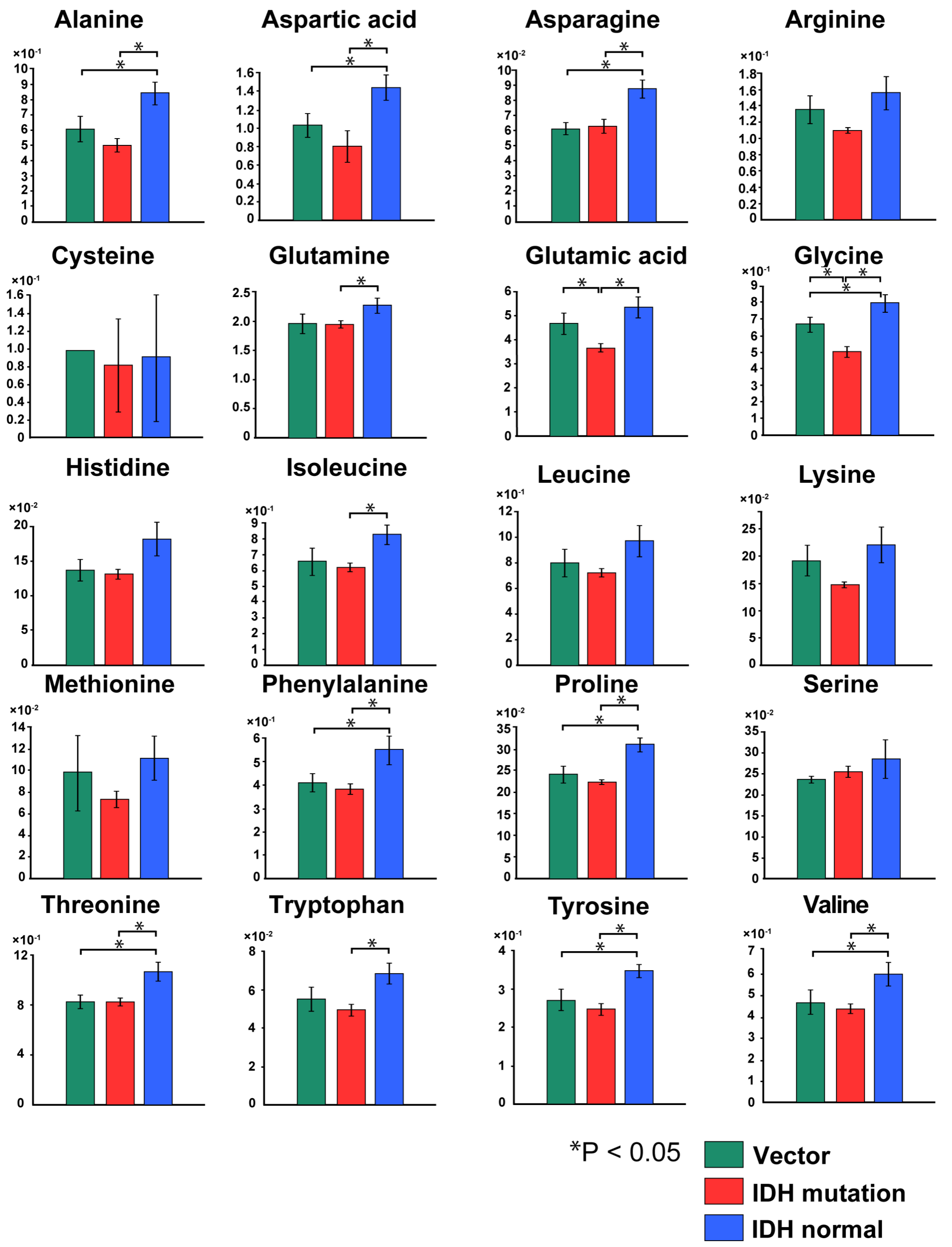

Supplementary Figure S2. Amino acid analysis in the cell experiment.

Amino acid production was reduced in the *IDH* mutant glioma cell line (red bars). Each vertical scale shows the relative quantification based on the internal standard. Green bars, empty vector-transfected cells; red bars, *IDH1*^{R132H}-transfected cells; blue bars, normal *IDH1*-transfected cells; error bar, standard error of mean. *P < 0.05.

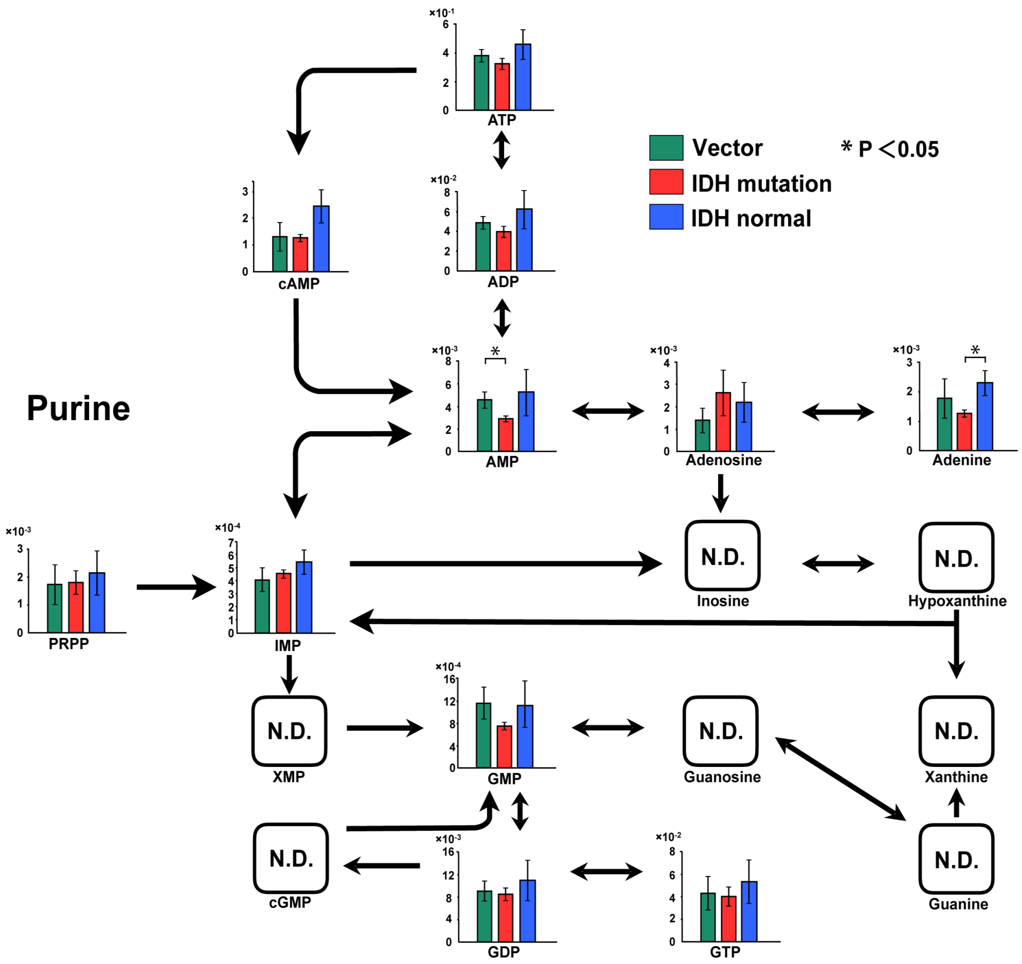

Supplementary Figure S3. Purine metabolic pathway in the cell experiment.
Purine metabolic production was reduced in the cells expressing *IDH* mutant protein (red bars). Each vertical scale shows the relative quantification based on the internal standard. Green bars, empty vector-transfected cells; red bars, *IDH1*^{R132H}-transfected cells; blue bars, normal *IDH1*-transfected cells; N.D., not detected; error bar, standard error of mean. *P < 0.05.

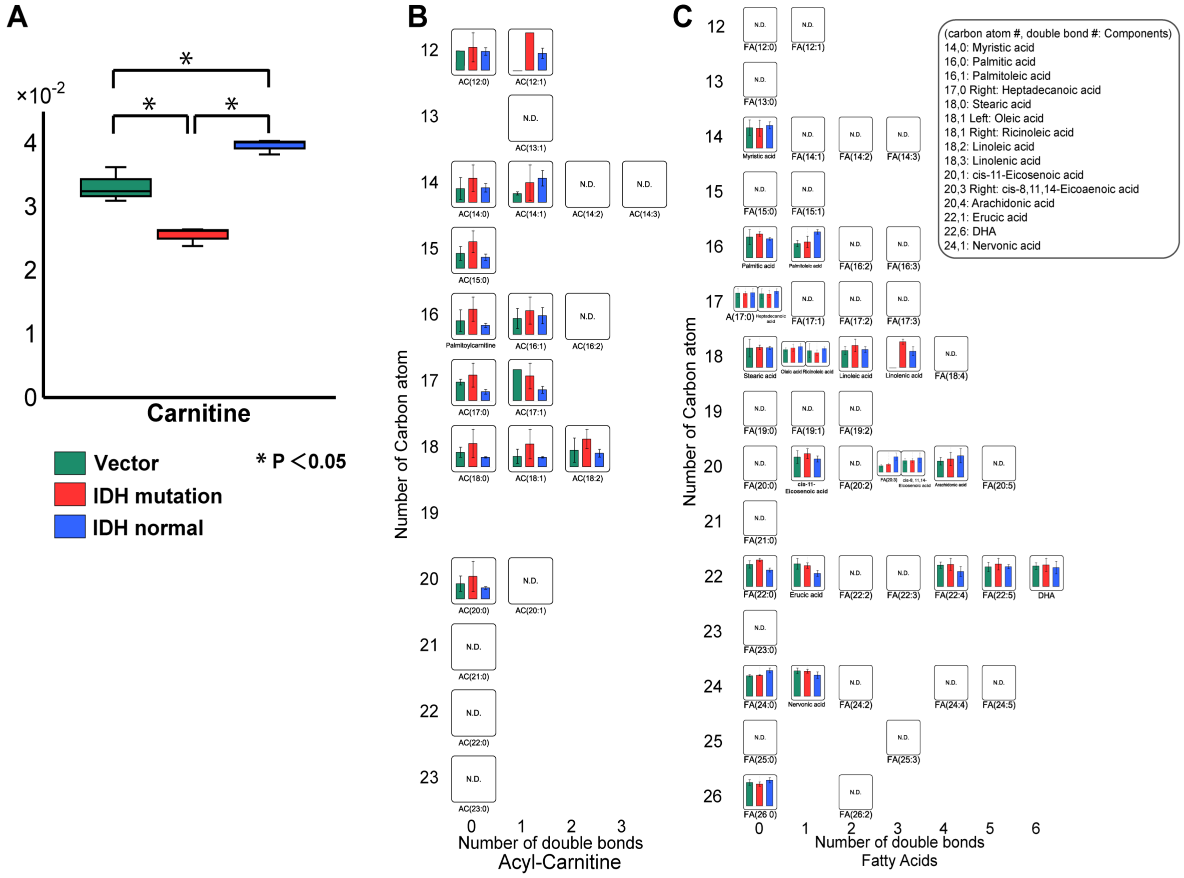

Supplementary Figure S4. Analysis of lipid metabolism in the cell experiment. (**A**) Carnitine was reduced in the mutant *IDH* expressing cells (red bars). (**B**) Acyl-carnitine, which was required the same or slightly elevated in the cells expressing *IDH* mutant protein (red bars). (**C**) Fatty acid production tended to be enhanced in the cells expressing *IDH* mutant protein (red bars). The numeric labels on the left side, horizontal axis, and vertical axis represent the number of carbon atoms, the number of double bonds, and the relative quantification based on the internal standard, respectively. Green bars, empty vector-transfected cells; red bars, *IDH1*^{R132H}-transfected cells; blue bars, *IDH1* normal-transfected cells, N.D., not detected; error bar, standard error of mean. *P < 0.05.

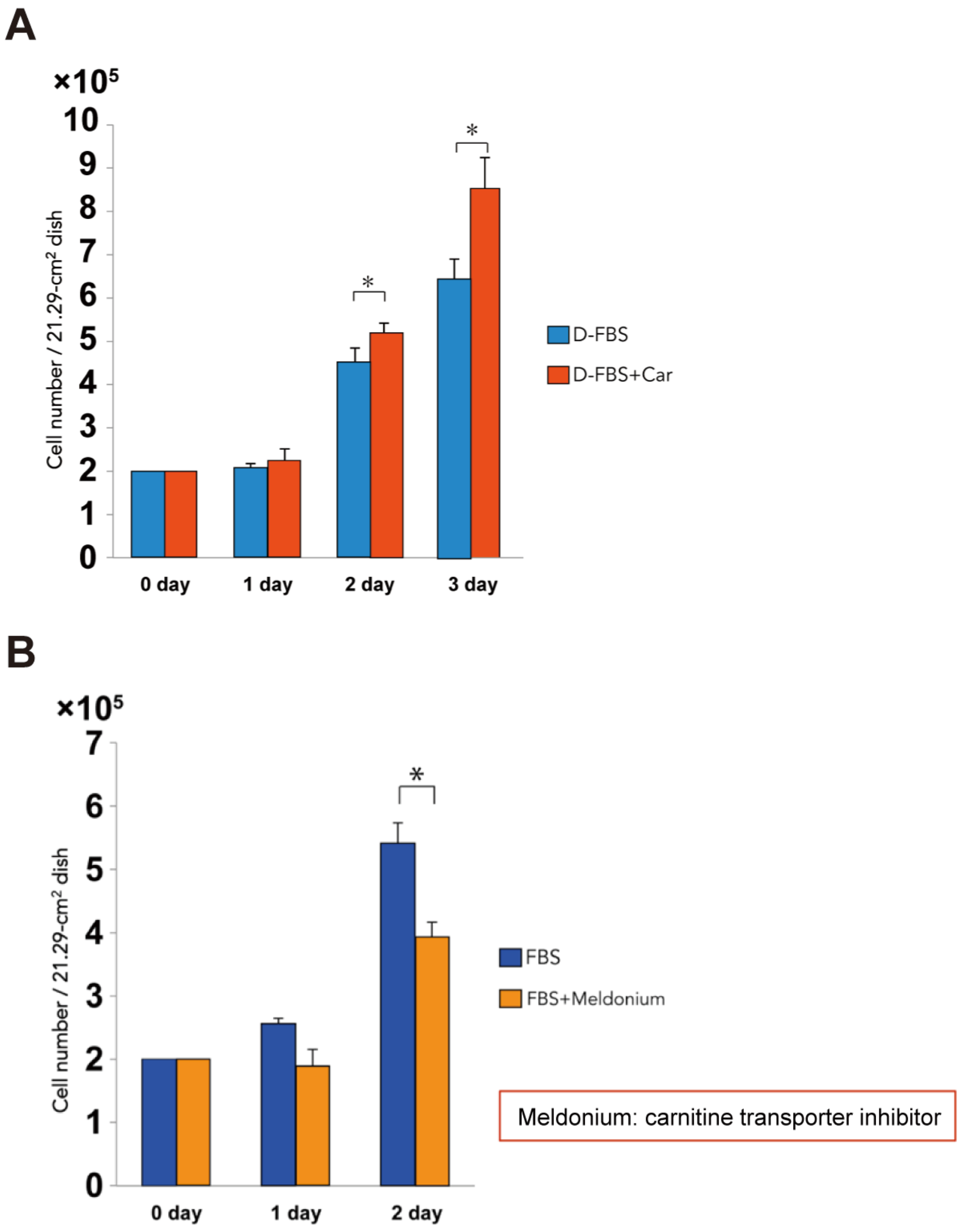

Supplementary Figure S5. Proliferation analysis of U87 cells in culture medium with or without carnitine and meldonium.

(A) U87 cells (2 x10^5 cells) were plated on 60-mm (21.29-cm^2) culture dishes. Cells were cultured in D-MEM containing with 10% dialyzed fetal bovine serum (D-FBS) with (D-FBS + Car) or without 100 μM carnitine. The number of cells was counted everyday. (B) Cells were either incubated in D-MEM containing with 10% fetal bovine serum (FBS) or FBS supplemented with 2 mM mildronate (FBS + Meldonium). error bar, standard error of mean. *$P < 0.05$.

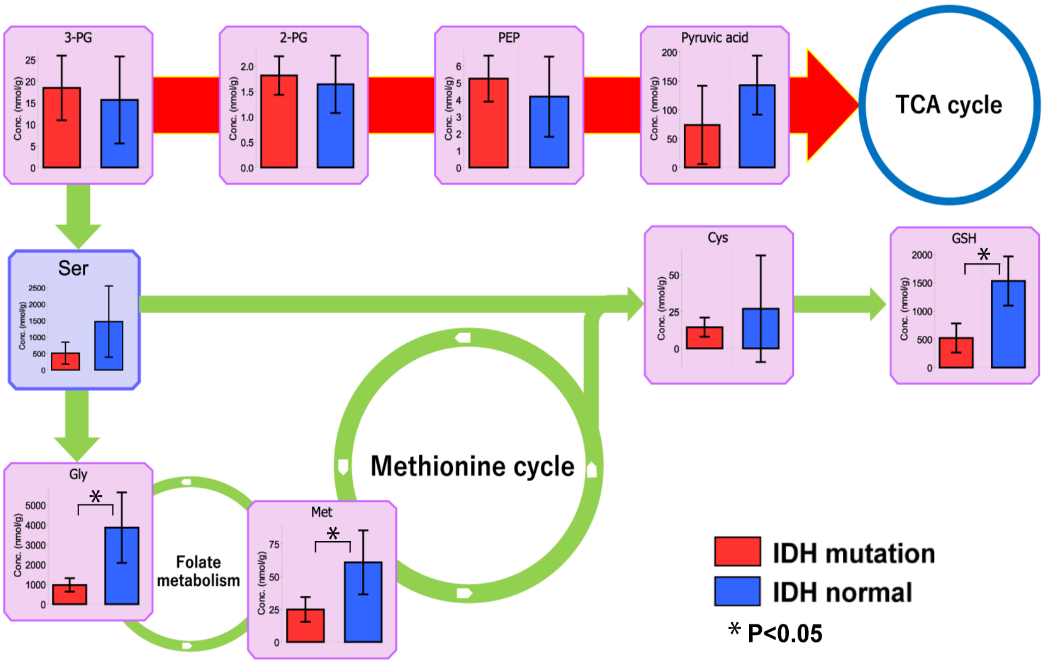

Supplementary Figure S6. Analysis of serine, folate and methionine in *IDH* mutation gliomas tissues.

Serine, glycine, and methionine were decreased in gliomas tissues with *IDH* mutation (Fig. 2C). The decrease of serine results in the suppression of folate metabolism and glutathione production. Folate is related to cell division and glutathione is related to oxidative stress. These results may also link to the better prognosis of gliomas with the *IDH* mutation. The vertical scale shows the absolute quantification (nmol/g). Red bars, *IDH1*^{R132H} glioma; blue bars, normal *IDH1* glioma. standard error of mean. *P < 0.05.

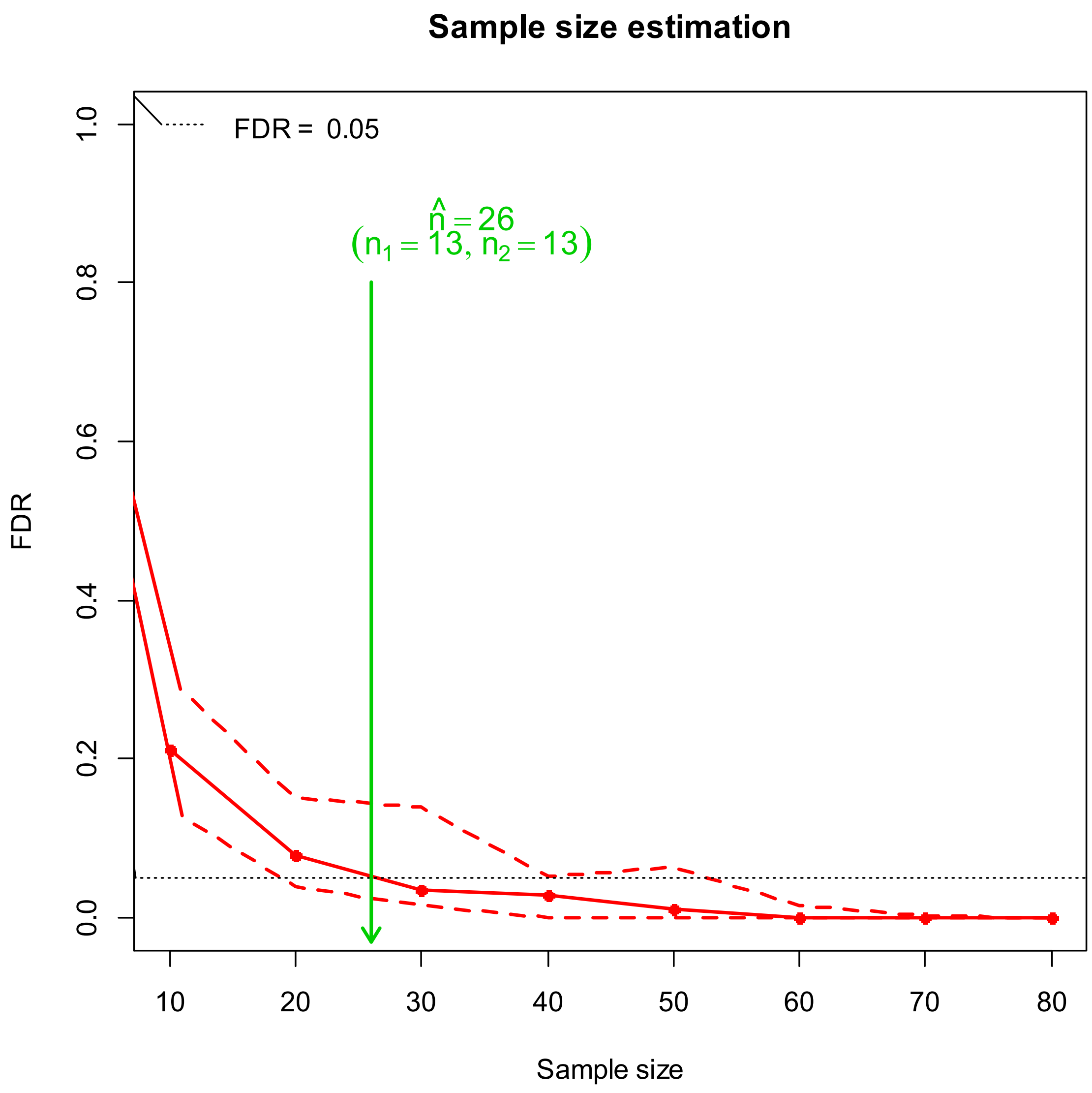

Supplementary Figure S7. Sample size estimation without target pilot data using probabilistic principal components analysis model.

We examined the appropriate number of clinical samples using MetSizeR. The analysis condition was the follows, without pilot targeted data, metabolites=100, proportion of significant metabolites=0.3, probabilistic principal components analysis model, target FDR (false discovery rate) = 0.05, sample size per group = 5. This condition is similar to our experimental condition. Solid red line, the estimated FDR; dashed red lines, the 10th and 90th percentiles; horizontal dashed black line, the target FDR at 5%.

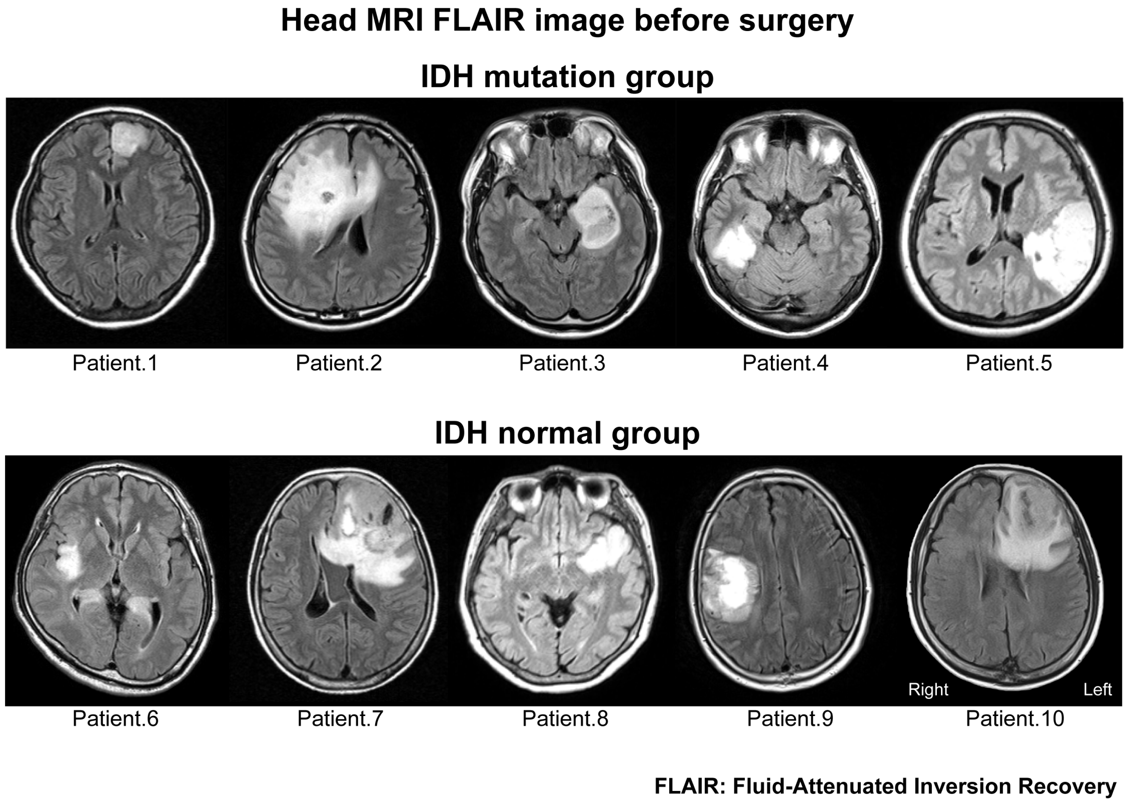

Supplementary Figure S8. Head MRI FLAIR image before surgery.
Patient 1-5 are *IDH* mutation group (Patient 1; Oligodendroglioma, Patient 2; Oligodendroglioma, Patient 3; Anaplastic Astrocytoma, Patient 4; Anaplastic Astrocytoma, Patient 5; Anaplastic Oligodendroglioma). Patient 6-10 are *IDH* normal group (Patient 6; Diffuse Astrocytoma, Patient 7; Anaplastic Astrocytoma, Patient 8; Anaplastic Astrocytoma, Patient 9; Glioblastoma; Patient 10; Glioblastoma). FLAIR, Fluid-attenuated inversion recovery.

Supplementary Table S1

Two-Sample T-Tests Assuming Equal Variance for carnitine in clinical samples using PASS16

Numeric Results for Two-Sample T-Test Assuming Equal Variance

Alternative Hypothesis: H1: δ = μ1 - μ2 ≠ 0

Power	N1	N2	N	δ	σ	Alpha
**0.70378**	**5**	**5**	**10**	**1.7**	**0.4**	**$0.00033=1-(0.95)^{1/151}$ ≈0.05/151(Bonferroni correction)**
0.99994	5	5	10	1.7	0.4	0.05

Report Definitions

Power is the probability of rejecting a false null hypothesis.

N1 and N2 are the number of items sampled from each population.

N is the total sample size, N1 + N2.

μ1 and μ2 are the assumed population means.

δ = μ1 - μ2 is the difference between population means at which power and sample size calculations are made.

σ is the assumed population standard deviation for each of the two groups.

Alpha is the probability of rejecting a true null hypothesis.

Summary Statements

Group sample sizes of 5 and 5 achieve 70.378% power to reject the null hypothesis of equal means when the population mean difference is 1.7 with a standard deviation for both groups of 0.4 and with a significance level (alpha) of 0.00033 using a two-sided two-sample t-test.

Supplementary. Table S2

Patient information

Patient	WHO grade	*IDH* state	Tumor	Age (years)	Sex
Pt 1	II	R132H	Oligodendroglioma	53	F
Pt 2	II	R132H	Oligodendroglioma	38	M
Pt 3	III	R132H	Anaplastic Astrocytoma	39	M
Pt 4	III	R132H	Anaplastic Astrocytoma	40	M
Pt 5	III	R132H	Anaplastic Oligodendroglioma	52	F
Pt 6	II	normal type	Diffuse Astrocytoma	59	M
Pt 7	III	normal type	Anaplastic Astrocytoma	64	F
Pt 8	III	normal type	Anaplastic Astrocytoma	59	F
Pt 9	IV	normal type	Glioblastoma	62	M
Pt 10	IV	normal type	Glioblastoma	66	F

IDH, isocitrate dehydrogenase; F, female; M, male

Five patients each were in the *IDH* mutant and *IDH* normal groups. The *IDH* mutations were all R132H.

**Patient 1**	53 years old	female
brain tumor name	Oligodendroglioma	
Anamnesis	Nothing	
Preoperative oral medicine	phenytoin 200 mg/day	
Preoperative blood counts	White blood cell counts	4400 /μL
	Red blood cell counts	389×10^4 /μL
	Hemoglobin	12.4 g/dL
	Platelet counts	28.1×10^4 /μL
Preoperative blood biochemical examination	Total protein	7.7 g/dL
	Urea nitrogen	10 mg/dL
	Creatinine	0.58 mg/dL
	Total bilirubin	1.02 mg/dL
	GOT (glutamic oxaloacetic transaminase)	15 mU/mL
	GPT (glutamic pyruvic transaminase)	7 mU/mL
	CPK (creatine phosphokinase)	84 mU/mL
	Sodium	140 mmol/L
	Potassium	4.1 mmol/L
	Chlorine	105 mmol/L
	Blood sugar	93 mg/dL

**Patient 2**	38 years old	male
brain tumor name	Oligodendroglioma	
Anamnesis	Nothing	
Preoperative oral medicine	carbamazepine 400 mg/day, betamethasone 3 mg/day, famotidine 40 mg/day	
Preoperative blood counts	White blood cell counts	10400 /μL
	Red blood cell counts	473×10^4 /μL
	Hemoglobin	13.9 g/dL
	Platelet counts	25.2×10^4 /μL
Preoperative blood biochemical examination	C-reactive protein	0.02 mg/dL
	Total protein	6.5 g/dL
	Albumin	3.8 g/dL
	Urea nitrogen	17 mg/dL
	Creatinine	0.63 mg/dL
	Total bilirubin	0.70 mg/dL
	GOT (glutamic oxaloacetic transaminase)	36 mU/mL
	GPT (glutamic pyruvic transaminase)	148 mU/mL
	LDH (lactate dehydrogenase)	266 mU/mL
	CPK (creatine phosphokinase)	211 mU/mL
	Sodium	136 mmol/L
	Potassium	4.5 mmol/L
	Chlorine	100 mmol/L

**Patient 3**	39 years old	male
brain tumor name	Anaplastic Astrocytoma	
Anamnesis	Nothing	
Preoperative oral medicine	carbamazepine 400 mg/day	
Preoperative blood counts	White blood cell counts	4000 /μL
	Red blood cell counts	473×10^4 /μL
	Hemoglobin	14.0 g/dL
	Platelet counts	24.2×10^4 /μL
Preoperative blood biochemical examination	Total protein	7.0 g/dL
	Albumin	4.5 g/dL
	Urea nitrogen	12 mg/dL
	Creatinine	0.70 mg/dL
	Uric acid	5.3 mg/dL
	Total bilirubin	0.56 mg/dL
	GOT (glutamic oxaloacetic transaminase)	17 mU/mL
	GPT (glutamic pyruvic transaminase)	22 mU/mL
	LDH (lactate dehydrogenase)	166 mU/mL
	ALP (alkaline phosphatase)	216 mU/mL
	γGT (γ guru Tamil transformer peptidase)	56 mU/mL
	Sodium	141 mmol/L
	Potassium	4.4 mmol/L
	Chlorine	104 mmol/L
	Total cholesterol	154 mg/dL
	Triglyceride	94 mg/dL
	Cholinesterase	289 mU/mL

**Patient 4**	40 years old	male
brain tumor name	Anaplastic Astrocytoma	
Anamnesis	Nothing	
Preoperative oral medicine	carbamazepine 400 mg/day	
Preoperative blood counts	White blood cell counts	7500 /μL
	Red blood cell counts	493×10^4 /μL
	Hemoglobin	15.9 g/dL
	Platelet counts	29.5×10^4 /μL
Preoperative blood biochemical examination	Total protein	7.4 g/dL
	Urea nitrogen	16 mg/dL
	Creatinine	0.74 mg/dL
	Total bilirubin	0.39 mg/dL
	GOT (glutamic oxaloacetic transaminase)	32 mU/mL
	GPT (glutamic pyruvic transaminase)	51 mU/mL
	CPK (creatine phosphokinase)	93 mU/mL
	Sodium	143 mmol/L
	Potassium	4.1 mmol/L
	Chlorine	106 mmol/L
	Blood sugar	150 mg/dl

**Patient 5**	52 years old	female
brain tumor name	Anaplastic Oligodendroglioma	
Anamnesis	Reflux esophagitis	
Preoperative oral medicine	sodium valproate 800 mg/day, lansoprazole 30 mg/day	
Preoperative blood counts	White blood cell counts	7400 /μL
	Red blood cell counts	484×10^4 /μL
	Hemoglobin	14.7 g/dL
	Platelet counts	24.0×10^4 /μL
Preoperative blood biochemical examination	Total protein	7.6 g/dL
	Albumin	4.9 g/dL
	Urea nitrogen	8 mg/dL
	Creatinine	0.49 mg/dL
	Total bilirubin	1.95 mg/dL
	GOT (glutamic oxaloacetic transaminase)	20 mU/mL
	GPT (glutamic pyruvic transaminase)	28 mU/mL
	LDH (lactate dehydrogenase)	154 mU/mL
	γGT (γ guru Tamil transformer peptidase)	30 mU/mL
	CPK (creatine phosphokinase)	86 mU/mL
	Sodium	145 mmol/L
	Potassium	3.2 mmol/L
	Chlorine	106 mmol/L
	Blood sugar	106 mg/dL

**Patient 6**	59 years old	male
brain tumor name	Diffuse Astrocytoma	
Anamnesis	Nothing	
Preoperative oral medicine	sodium valproate 800 mg/day	
Preoperative blood counts	White blood cell counts	4800 /μL
	Red blood cell counts	401×10^4 /μL
	Hemoglobin	12.8 g/dL
	Platelet counts	23.7×10^4 /μL
Preoperative blood biochemical examination	Total protein	5.5 g/dL
	Albumin	3.6 g/dL
	Urea nitrogen	14 mg/dL
	Creatinine	0.80 mg/dL
	Total bilirubin	0.94 mg/dL
	GOT (glutamic oxaloacetic transaminase)	13 mU/mL
	GPT (glutamic pyruvic transaminase)	10 mU/mL
	LDH (lactate dehydrogenase)	150 mU/mL
	ALP (alkaline phosphatase)	263 mU/mL
	γGT (γ guru Tamil transformer peptidase)	10 mU/ml
	CPK (creatine phosphokinase)	57 mU/mL
	Sodium	140 mmol/L
	Potassium	4.2 mmol/L
	Chlorine	108 mmol/L
	Blood sugar	76 mg/dL
	Total cholesterol	194 mg/dL
	Triglyceride	225 mg/dL
	Cholinesterase	559 mU/mL

**Patient 7**	64 years old	female
brain tumor name	Anaplastic Astrocytoma	
Anamnesis	Gastric polyp	
Preoperative oral medicine	magnesium oxide 1500 mg/day	
Preoperative blood counts	White blood cell counts	5100 /μL
	Red blood cell counts	423×10^4 /μL
	Hemoglobin	12.6 g/dL
	Platelet counts	25.6×10^4 /μL
Preoperative blood biochemical examination	Total protein	7.7 g/dL
	Urea nitrogen	16 mg/dL
	Creatinine	0.52 mg/dL
	Total bilirubin	0.62 mg/dL
	GOT (glutamic oxaloacetic transaminase)	16 mU/mL
	GPT (glutamic pyruvic transaminase)	11 mU/mL
	CPK (creatine phosphokinase)	47 mU/mL
	Sodium	138 mmol/L
	Potassium	3.8 mmol/L
	Chlorine	100 mmol/L
	Blood sugar	112 mg/dL

**Patient 8**	59 years old	female
brain tumor name	Anaplastic Astrocytoma	
Anamnesis	Cholelithiasis	
Preoperative oral medicine	sodium valproate 800 mg/day, Ursodeoxycholic acid 300mg/day, Furopuropion 240 mg/day	
Preoperative blood counts	White blood cell counts	4800 /μL
	Red blood cell counts	394×10^4 /μL
	Hemoglobin	12.2 g/dL
	Platelet counts	20.9×10^4 /μL
Preoperative blood biochemical examination	Total protein	6.8 g/dL
	Albumin	4.1 g/dL
	Urea nitrogen	14 mg/dL
	Creatinine	0.59 mg/dL
	Total bilirubin	1.54 mg/dL
	GOT (glutamic oxaloacetic transaminase)	12 mU/mL
	GPT (glutamic pyruvic transaminase)	8 mU/mL
	LDH (lactate dehydrogenase)	159 mU/mL
	ALP (alkaline phosphatase)	180 mU/mL
	γGT (γ guru Tamil transformer peptidase)	13 mU/mL
	CPK (creatine phosphokinase)	68 mU/mL
	Sodium	142 mmol/L
	Potassium	4.3 mmol/L
	Chlorine	106 mmol/L
	Blood sugar	85 mg/dL
	Cholinesterase	306 mU/mL

**Patient 9**	62 years old	male
brain tumor name	Glioblastoma	
Anamnesis	Hypertension	
Preoperative oral medicine	sodium valproate 800 mg/day, isosorbide 90 ml/day, candesartan 8 mg/day, azelnidipine 8 mg/day	
Preoperative blood counts	White blood cell counts	5500 /μL
	Red blood cell counts	471×10^4 /μL
	Hemoglobin	15.0 g/dL
	Platelet counts	14.3×10^4 /μL
Preoperative blood biochemical examination	C-reactive protein	0.06 mg/dL
	Total protein	6.7 g/dL
	Albumin	4.0 g/dL
	Urea nitrogen	22 mg/dL
	Creatinine	0.94 mg/dL
	Total bilirubin	1.06 mg/dL
	Direct bilirubin	0.14 mg/dL
	GOT (glutamic oxaloacetic transaminase)	26 mU/mL
	GPT (glutamic pyruvic transaminase)	38 mU/mL
	LDH (lactate dehydrogenase)	165 mU/mL
	γGT (γ guru Tamil transformer peptidase)	27 mU/mL
	Sodium	143 mmol/L
	Potassium	4.0 mmol/L
	Chlorine	108 mmol/L
	Blood sugar	104 mg/dL

**Patient 10**	66 years old	female
brain tumor name	Glioblastoma	
Anamnesis	Emphysema, Gastric ulcer	
Preoperative oral medicine	phenytoin 200 mg/day, theophylline 200 mg/day, porapurejinku 150 mg/day	
Preoperative blood counts	White blood cell counts	5100 /μL
	Red blood cell counts	406×10^4 /μL
	Hemoglobin	12.2 g/dL
	Platelet counts	18.9×10^4 /μL
Preoperative blood biochemical examination	Total protein	7.1 g/dL
	Albumin	3.8 g/dL
	Urea nitrogen	16 mg/dL
	Creatinine	0.67 mg/dL
	Total bilirubin	0.51 mg/dL
	GOT (glutamic oxaloacetic transaminase)	21 mU/mL
	GPT (glutamic pyruvic transaminase)	16 mU/mL
	LDH (lactate dehydrogenase)	171 mU/mL
	ALP (alkaline phosphatase)	246 mU/mL
	γGT (γ guru Tamil transformer peptidase)	18 mU/mL
	Sodium	143 mmol/L
	Potassium	4.2 mmol/L
	Chlorine	105 mmol/L
	Blood sugar	87 mg/dL

Supplementary. Table S3

A

	cell amount ($\times 10^6$ cells)	
sample name	CE-TOFMS	LC-TOFMS
GFP 1	4.63	5.44
GFP 2	4.63	5.44
GFP 3	4.63	5.44
IDH mutation 1	4.45	3.68
IDH mutation 2	4.45	3.68
IDH mutation 3	4.45	3.68
IDH wild 1	4.05	3.41
IDH wild 2	4.05	3.41
IDH wild 3	4.05	3.41

CE-TOFMS: capillary electrophoresis mass spectrometry with time-of-flight

LC-TOFMS: liquid chromatography time-of-flight mass spectrometry

GFP: Green Fluorescent Protein

B

	sample amount (mg)	
patient number	CE-TOFMS	LC-TOFMS
patient 1	12	5
patient 2	55	74
patient 3	48	44
patient 4	41	79
patient 5	64	94
patient 6	25	67
patient 7	31	27
patient 8	39	73
patient 9	86	80
patient 10	23	42

CE-TOFMS: capillary electrophoresis mass spectrometry with time-of-flight

LC-TOFMS: liquid chromatography time-of-flight mass spectrometry

Supplementary. Table S4

The conditions for CE-TOFMS and LC-TOFMS made in Human Metabolome Technologies Inc.

A

CE-TOFMS

Agilent CE-TOFMS system (Agilent Technologies)

Capillary: Fused silica capillary i.d. 50 μm × 80 cm

	cation mode	anion mode
Run buffer	Cation Buffer Solution (p/n:H3301-1001)	Anion Buffer Solution (p/n:H3302-1021)
Rinse buffer	Cation Buffer Solution (p/n:H3301-1001)	Anion Buffer Solution (p/n:H33022-1022)
Sample injection	Pressure injection 50mbar, 10 sec	Pressure injection 50mbar, 25 sec
CE voltage	Positive, 27kV	Positive, 30kV
MS ionization	ESI Positive	ESI Negative
MS capillary voltage	4000V	3500V
MS scan range	m/z 50-1000	m/z 50-1000
Sheath liquid	HMT Sheath Liquid (p/n: H3301-1020)	HMT Sheath Liquid (p/n: H3301-1020)

B

LC-TOFMS

LC system	Agilent 1200 series Rapid Resolution LC system SL (Agilent Technologies)	
Column	Octa Decyl Silyl column, 2 × 50 mm, 2 μm	
MS system	Agilent LC/MSD TOF (Agilent Technologies)	

	positive mode	negative mode
column temperature	40℃	40℃
Mobile phase	A: H_2O/0.1% HCOOH<br>B: Isopropanol: Acetonitrile: H_2O (65:30:5)/0.1% HCOOH, 2mM $HCOONH_4$	A: H_2O/0.1% HCOOH<br>B: Isopropanol: Acetonitrile: H_2O (65:30:5)/0.1% HCOOH, 2mM $HCOONH_4$
Flow rate	0.3 mL/min	0.3 mL/min
Run time	20 min	20 min
Post time	6 min	6 min
Gradient condition	0-0.5 min: B 1%<br>0.5-13.5 min: B 1-100%<br>13.5-20 min: B 100%	0-0.5 min: B 1%<br>0.5-13.5 min: B 1-100%<br>13.5-20 min: B 100%
MS ionization mode	ESI Positive	ESI Negative
MS Nebulizer pressure	40 psi	40 psi
MS dry gas flow	10 L/min	10 L/min
MS dry gas temperature	350℃	350℃
MS capillary voltage	4000 V	4000 V
MS scan range	m/z 100-1700	m/z 100-1700

Supplementary. Table S5

List of Abbreviations (Figure, table, supplementary Figure, supplementary table)

List of Abbreviations	Compound name
1,3-Diaminopropane	1,3-Diaminopropane
Methylimidazoleacetic acid	1-Methyl-4-imidazoleacetic acid
Pyrroline 5-carboxylic acid	1-Pyrroline 5-carboxylic acid
Diphosphoglycerate	2,3-Diphosphoglyceric acid
Gensigen	2,5-Dihydroxybenzoic acid
dAdenosine	2'-Deoxyadenosine
dCyt	2'-Deoxycytidine
dGuanosine	2'-Deoxyguanosine
dR1P	2-Deoxyribose 1-phosphate
dUri	2'-Deoxyuridine
2-OG	2-Oxoglutaric acid
2-KIV	2-Oxoisovaleric acid
Phenylethylamine	2-Phenylethylamine
2-PG	2-Phosphoglyceric acid
T3	3,3',5-Triiodothyronine
Dephospho-CoA	3'-Dephospho CoA
3-OHAA	3-Hydroxyanthranilic acid
b-Lactate	3-Hydroxypropionic acid
MIT	3-Iodotyrosine
MHPG	3-Methoxy-4-hydroxyphenylethyleneglycol
2K3MVA	3-Methyl-2-oxovaleric acid
3-PG	3-Phosphoglyceric acid
2-Oxoleucine	4-Methyl-2-oxovaleric acid
KMTB	4-Methylthio-2-oxobutyric acid
5-Aminolevulinic acid	5-Amino-4-oxovaleric acid
AICAR	5-Aminoimidazole-4-carboxamide ribotide
MTA	5'-Deoxy-5'-methylthioadenosine
5-Hydroxy-IAA	5-Hydroxyindoleacetic acid
Pretonine	5-Hydroxytryptophan
5MOT	5-Methoxytryptamine
Oxoproline	5-Oxoproline
6-PG	6-Phosphogluconic acid
AcCoA	Acetyl CoA_divalent
Succinyl AMP	Adenylosuccinic acid

ADP	Adenosine diphosphate
ADP-Rib	Adenosine diphosphate-ribose
Ala	Alanine
AMP	Adenosine monophosphate
Arg	Arginine
ArgSuccinate	Argininosuccinic acid
Asn	Asparagine
Asp	Aspartic acid
ATP	Adenosine Triphosphate
BTL	Betaine aldehyde_+H_2O
cAMP	cyclic Adenosine monophosphate
Carbamoyl-P	Carbamoylphosphate
CDP	Cytidine dimonophosphate
CDP-choline	Cytidine dimonophosphate-choline
cGMP	Cyclic guanosine monophosphate
CMP	cytidine monophosphate
CMP-NeuNAc	cytidine monophosphate-*N*-acetylneuraminate
CoA	Coenzyme A_divalent
CTP	Cytidine triphosphate
Cys	Cysteine
Cys-Gly	Cysteine-Glycine
dADP	deoxyadenosine 5'-diphosphate
dAMP	deoxyadenosine monophosphate
dATP	deoxyadenosine 5'-triphosphate
dCDP	deoxycytidine 5'-diphosphate
dCMP	deoxycytidine monophosphate
dCTP	deoxycytidine 5'-triphosphate
Deamido-NAD	Deamido-nicotinamide adenine dinucleotide+
dGDP	deoxyguanosine 5'-diphosphate
dGMP	deoxyguanosine monophosphate
dGTP	deoxyguanosine 5'-triphosphate
DHAP	Dihydroxyacetone phosphate
dIMP	deoxyinosine monophosphate
dITP	deoxyinosine triphosphate
DOPA	deoxyphenylalanine
dTDP	deoxythymidine diphosphate
TDP-Glc	deoxythymidine diphosphate -glucose
dTMP	deoxythymidine monophosphate

dTTP	deoxythymidine 5'-triphosphate
dUDP	deoxyuridine diphosphate
dUMP	deoxyuridine monophosphate
dUTP	deoxyuridine triphosphate
E4P	Erythrose 4-phosphate
EAP	Ethanolamine phosphate
F1,6P	Fructose 1,6-diphosphate
D-F1P	Fructose 1-phosphate
F6P	Fructose 6-phosphate
GABA	g-Aminobutyric acid
Gal1P	Galactose 1-phosphate
GDP	guanosine diphosphate
GDP-fucose	guanosine diphosphate-fucose
GDP-Man	guanosine diphosphate-mannose
Gln	Glutamine
Glu	Glutamic acid
Glc-6P	Glucosamine 6-phosphate
G1P	Glucose 1-phosphate
G6P	Glucose 6-phosphate
Glutaryl-CoA	Glutaryl CoA_divalent
GSH	Glutathione
GSSG	Glutathione_divalent
Gly	Glycine
GPCho	Glycerophosphocholine
Glycocholic acid	Glycocholic acid
Glycolic acid	Glycolic acid
GMP	guanosine monophosphate
GTP	guanosine triphosphate
His	Histidine
IDP	inosine diphosphate
Ile	Isoleucine
IAA	Imidazole-4-acetic acid
IMP	inosine monophosphate
Indoleacetaldehyde	Indole-3-acetaldehyde
3-IAA	Indole-3-acetic acid
Isobutyryl-CoA	Isobutyryl CoA_divalent
ITP	inosine triphosphate
Leu	Leucine

Lys	Lysine
Malic acid	Malic acid
Malonyl-CoA	Malonyl CoA_divalent
Man1P	Mannose 1-phosphate
Man6P	Mannose 6-phosphate
Met	Methionine
Methylmalonic acid	Methylmalonic acid
Myoinositol 1-phosphate	*myo*-Inositol 1-phosphate
Myoinositol 3-phosphate	*myo*-Inositol 3-phosphate
DMG	*N*,*N*-Dimethylglycine
N1-Methyl-4-pyridone-5-carboxamide	N^1-Methyl-4-pyridone-5-carboxamide
Trimethyllysine	N^6,N^6,N^6-Trimethyllysine
N-Acetylaspartic acid	*N*-Acetylaspartic acid
GlcNAc	*N*-Acetylglucosamine
GlcNAc-P	*N*-Acetylglucosamine 1-phosphate
NAcGlcNP	*N*-Acetylglucosamine 6-phosphate
N-AcGlu	*N*-Acetylglutamic acid
NeuNAc	*N*-Acetylneuraminic acid
N-AcOrn	*N*-Acetylornithine
N-Acetylputrescine	*N*-Acetylputrescine
NAD+	nicotinamide adenine dinucleotide+
NADP+	nicotinamide adenine dinucleotide phosphate+
Carbamoyl-Asp	*N*-Carbamoylaspartic acid
N-Formyl aspartic acid	*N*-Formylaspartic acid
Dipterine	*N*-Methyltryptamine
N-Methyltyramine	*N*-Methyltyramine
ALCAL	*O*-Acetylcarnitine
2-Aminophenol	*o*-Aminophenol
2-HPAA	*o*-Hydroxyphenylacetic acid
3PSer	*O*-Phosphoserine
Orotidine5'P	Orotidine 5'-monophosphate
ApppppA	P^1, P^4-Di(adenosine-5') tetraphosphate_divalent
Phe	Phenylalanine
Phenylpyruvate	Phenylpyruvic acid
PEP	Phosphoenolpyruvic acid
4-Hydroxyphenylacetic acid	*p*-Hydroxyphenylacetic acid
HPP	*p*-Hydroxyphenylpyruvic acid
Phytic acid	Phytic acid_divalent

Pro	Proline
Propanoyl-CoA	Propionyl CoA_divalent
PRPP	phosphoribosyl pyrophosphate
R1P	Ribose 1-phosphate
R5P	Ribose 5-phosphate
Ru5P	Ribulose 5-phosphate
SAHC	*S*-Adenosylhomocysteine
SAM	*S*-Adenosylmethionine
S7P	Sedoheptulose 7-phosphate
Ser	Serine
SucCoA	Succinyl CoA_divalent
Thr	Threonine
4-L-Hydroxyproline	*trans*-4-Hydroxyproline
Trp	Tryptophan
Tyr	Tyrosine
UDP	uridine 5'-diphosphate
UDP-Gal	uridine 5'-diphosphate-galactose
UDP-Glc	uridine 5'-diphosphate-glucose
UDP-GlcA	uridine 5'-diphosphate-glucuronic acid
UDP-GlcNAc	uridine 5'-diphosphate-*N*-acetylglucosamine
UMP	uridine monophosphate
UTP	uridine triphosphate
Val	Valine
VMA	Vanillylmandelic acid
XMP	xanthylic acid
XTP	XTP
X5P	Xylulose 5-phosphate
b-Ala	β-Alanine
Actinine	γ-Butyrobetaine
g-Glu-Cys	γ-Glutamic acid-Cysteine
